# Novel mechanism whereby metformin improves glucose homeostasis: TXNIP–GLUT1 axis modulation enhances intestinal glucotonic effects

**DOI:** 10.1038/s12276-025-01518-w

**Published:** 2025-08-06

**Authors:** Chan Woo Kang, Jung Ho Nam, Ju Hun Oh, Eun Kyung Wang, Soo Hyun Lee, Hye Ju Shin, Ye Bin Kim, Eun Jig Lee, Byung Kook Lim, Sungsoon Fang, Arthur Cho, Cheol Ryong Ku

**Affiliations:** 1 Endocrinology, Institute of Endocrine Research, Department of Internal Medicine, Yonsei University College of Medicine, Seoul, Republic of Korea; 2 Department of Internal Medicine, Graduate School of Medical Science, Brain Korea 21 Project, Yonsei University College of Medicine, Seoul, Republic of Korea; 3 Neurobiology Section, Division of Biological Sciences, University of California, San Diego, La Jolla, CA USA; 4 Department of Biomedical Sciences, Gangnam Severance Hospital, Yonsei University College of Medicine, Seoul, Republic of Korea; 5 Department of Nuclear Medicine, Yonsei University College of Medicine, Seoul, Republic of Korea

**Keywords:** Endocrine system and metabolic diseases, Membrane trafficking, Mechanisms of disease

## Abstract

Metformin is widely used as a first-line therapy for type 2 diabetes mellitus. However, the molecular mechanisms by which it modulates intestinal glucose metabolism remain incompletely defined. Here metformin was orally administered to male C57BL/6 mice, followed by intraperitoneal glucose tolerance testing and fluorine-18 fluorodeoxyglucose tracing to evaluate glucose homeostasis. To investigate changes in intestinal glucose metabolism, IEC6 and Caco-2 cell lines were used for in vitro analysis, with organoid experiments conducted for further validation. qRT-PCR, western blotting, flow cytometry and immunohistochemistry were performed to elucidate the effects of metformin on glucose metabolism pathways. Metformin enhanced glucose uptake and excretion in the distal intestine, particularly in the ileum and colon. Mechanistically, metformin upregulated the expression and membrane localization of glucose transporter 1 (GLUT1) by downregulating thioredoxin-interacting protein (TXNIP) expression. Consistently, intestinal-specific overexpression of TXNIP abolished metformin-induced improvements in glucose tolerance, while pharmacological inhibition of GLUT1 similarly negated metformin’s glucose-lowering effects. Our findings identified intestinal glucose excretion, mediated through the intestinal TXNIP–GLUT1 regulatory axis, as a previously unrecognized contributor to metformin’s glucoregulatory action. These results highlight a novel intestinal mechanism underlying metformin’s efficacy and provide insights for potential therapeutic strategies beyond traditional glucose regulation.

## New insights into metformin’s role in glucose metabolism

Type 2 diabetes is a common health issue, and metformin is a widely used medication to manage it. Researchers explored how metformin affects glucose handling in the intestines. The researchers gave metformin to mice and observed increased glucose uptake and excretion in the intestines, particularly in the ileum and colon. They found that metformin enhances the activity of a protein called GLUT1, which helps transport glucose across cell membranes. This effect was linked to a decrease in another protein, TXNIP, which usually inhibits GLUT1. The study showed that metformin’s ability to lower blood sugar involves increasing GLUT1 activity by reducing TXNIP levels. This mechanism does not rely on AMPK (a protein involved in energy regulation), suggesting a new pathway for metformin’s action. These findings could lead to better diabetes treatments by targeting the intestinal TXNIP–GLUT1 pathway.

This summary was initially drafted using artificial intelligence, then revised and fact-checked by the author.

## Introduction

Type 2 diabetes and metabolic diseases are among the most prevalent health disorders in modern society, with a recent increase in the number of in-patient cases. Researchers have extensively studied metformin for its diverse pharmacological actions^1–3^. Researchers have proposed various functional mechanisms for metformin effects, and they regard the involvement of 5′-AMP-activated protein kinase (AMPK)-dependent pathways as pivotal^4–7^. Despite over six decades of clinical application and extensive research, the precise mode of action of metformin remains unclear.

Previous research focused on elucidating the glucose-lowering mechanisms of metformin in the adipose tissue, muscle and liver of diabetic models^8,9^. However, researchers lack a comprehensive understanding of its broad-spectrum glucose-lowering effects, including in nondisease models. Recent research has focused on the metabolic role of the intestine via exploring incretins, such as GLP-1^10,11^, microbiome^12,13^ and gut-derived peptides^10,14^. The potential metabolic benefits of metformin, attributed to its action in the intestine, have also been highlighted. Studies have reported a significant increase in metformin biodistribution in the intestine after oral administration^15^.

The solute carrier family 2 (SLC2) proteins or glucose transporters (GLUTs) are responsible for passive glucose transport across cell membranes^16^. Among them, GLUT1 (SLC2A1) is ubiquitously expressed and mediates basal glucose uptake in various tissues, including the intestine^17,18^. GLUT1 activity is closely regulated by its expression levels and membrane localization, both of which respond dynamically to cellular energy demand and metabolic stress.

Thioredoxin-interacting protein (TXNIP), a redox-sensitive regulator, binds and inhibits thioredoxin, thereby promoting oxidative stress and increasing intracellular reactive oxygen species (ROS)^19^. TXNIP expression is induced under hyperglycemic or oxidative conditions and has been implicated in insulin resistance, β-cell dysfunction and impaired glucose homeostasis^20–22^. Importantly, TXNIP negatively regulates glucose uptake by reducing membrane GLUT1 localization through internalization and degradation mechanisms^23,24^. While the GLUT1–TXNIP regulatory axis has been explored in various metabolic tissues, its role in the intestinal epithelium, especially in the context of metformin action, remains poorly defined.

Advanced imaging techniques such as positron emission tomography–computed tomography, have revealed that metformin stimulates intestinal glucose uptake^25,26^. Moreover, bariatric procedures, such as Roux-en-Y gastric bypass, enhance both intestinal glucose uptake and intraluminal glucose excretion, suggesting a dual role of the gut in systemic glucose regulation^27–29^. Although metformin influences intestinal glucose metabolism, most studies have focused on its uptake-promoting effects, and little is known about the mechanistic relevance of these changes in clinical glucose control.

In this study, we investigated the contribution of intestinal glucose handling to metformin’s systemic metabolic effects. Specifically, we identified the GLUT1–TXNIP axis as a key molecular pathway mediating metformin-induced alterations in intestinal glucose metabolism and propose that this mechanism plays a critical role in its glucose-lowering effect.

## Methods

### Animal models

C57BL/6 mice were purchased from Orientbio. All animal experiments followed the animal ethics guidelines and were approved by the Yonsei University Institutional Animal Care and Use Committee, Yonsei University School of Medicine (Seoul, Korea). Our study examined male mice because male animals exhibited less variability in phenotype. All animal handling procedures adhered to the guidelines established by Association for Assessment and Accreditation of Laboratory Animal Care International and were approved by Yonsei University Institutional Animal Care and Use Committee (permit no. 2022-0240).

### Animal experiments

After a 1-week acclimatization period, male C57BL/6 mice were orally gavaged with 150 mg/kg of metformin. The mice were fasted for 6 h, and an intraperitoneal glucose tolerance test (IPGTT) was performed. Metformin was gavaged 4 h before the start of the IPGTT. Fluorine-18 fluorodeoxyglucose (^18^F-FDG) injection was performed to trace glucose uptake. On the day of measurement, mice were orally administered metformin at a dose of 150 mg/kg 2 h after the start of fasting, and FDG was injected 2 h later. One hour after FDG injection, mice were euthanized, and the intestines were excised en bloc. Limbs were flushed with phosphate-buffered saline (PBS), and PBS washings were collected for gamma counting to quantify fecal FDG excretion. Autoradiography images of the post-wash intestines were obtained to identify areas of high glycolytic rates within the intestinal wall. Similar experimental methods were used to evaluate the glucose homeostasis effect of GLUT1 inhibition in metformin-treated mice. After a 1-week acclimatization period, mice were intraperitoneally injected with STF-31 (GLUT1-specific inhibitor) at a dose of 7.8 mg/kg once daily for 3 days. Three days later, mice received oral gavage of metformin at 150 mg/kg followed by injection of STF-31. Glucose homeostasis was assessed through an IPGTT with 2.5 g/kg glucose. The following day, mice received the same treatment of metformin and STF-31, followed by FDG injection and sacrifice, with subsequent sampling conducted as previously described.

### Establishment of the TXNIP-overexpressing diabetic animal model

C57BL/6, male mice were used in healthy mouse experiments or were rendered diabetic by multiple low-dose streptozotocin (STZ) injections (3 days; intraperitoneal injection; freshly prepared in 0.1 mmol/l sodium citrate at pH 4.5). Five days after the first STZ injection according to the in vivo transfection protocol (invivo-jetPEI, Polyplus), mice were subjected to tail vein injection of TXNIP. After TXNIP injection, metformin at a dose of 250 mg/kg was administered via oral gavage. On the fourth day of metformin treatment, an IPGTT was conducted. The following day, after administering metformin, mice were injected with ^18^F-FDG and euthanized 1 h later for further analysis. The fasting duration and the time intervals between metformin gavage and subsequent procedures on the IPGTT day, as well as on the FDG injection measurement day, were the same as in the previous metformin experiment.

### Cell culture

IEC6 cells were maintained at 37 °C under 5% CO_2_ in Dulbecco’s modified Eagle medium (Cytiva) supplemented with 10% fetal bovine serum and 1% penicillin–streptomycin. Caco-2 cells were grown in Minimal Essential Medium with Earle’s Balanced Salts (MEM/EBSS, Cytiva) medium supplemented with 10% fetal bovine serum and 1% penicillin–streptomycin and 25 mM HEPES.

### Crypt isolation and culture for organoid studies

Crypt isolation and culture for organoid studies the small intestines of C57BL/6 mice were collected. The fat and villi were removed, and the samples were incubated at 4 °C for 15 min to isolate the crypts. After passing the crypt suspension through a cell strainer and centrifugation, 50–200 crypts were suspended in 25 µl of Matrigel (#356231; BD Biosciences). From the isolation of intestinal crypt organoids to their maintenance and subsequent seeding into a monolayer for experimental use, procedures were conducted following protocols outlined in stem cell technology literature.

### 2DG uptake assay

2-Deoxy-d-glucose (2DG) uptake is performed according to the procedure described in the Glucose-Uptake-Glo Assay kit (Promega). Glucose uptake is processed for 60 min and is divided into three or four parts. Fluorescence was measured 60 min after treatment with 2-deoxyglucose 6-phosphate using GloMax discover (Promega). 2DG6P levels were normalized using the 3-(4,5-dimethylthiazol-2-yl)-5-(3-carboxymethoxyphenyl)-2-(4-sulfophenyl)-2H-tetrazolium(MTS) assay, which reflects cell viability.

### Glucose excretion

Cells were seeded in the upper wells of transwell inserts and allowed to form a monolayer. After 16 h of treatment with metformin, the upper wells were supplemented with high-glucose medium, while the lower wells received no glucose medium. After 30 min, glucose transport across the membrane was assessed using glucose colorimetric detection. To confirm whether glucose diffusion into the lower well occurred solely through passive diffusion or via cellular transport, dextran was utilized.

### RNA purification and quantitative PCR

Total RNA was isolated from cells and tissues using the RNeasy Mini Purification Kit (Qiagen) according to the manufacturer’s protocol. Tissues were homogenized in RLT buffer (QIAGEN), a lysis buffer that inactivates RNases and facilitates RNA extraction, using a bead homogenizer for 3 min at maximum speed (Qiagen, TissueLyser II). cDNA was prepared using ReverTra Ace (Toyobo) according to the manufacturer’s instructions. The resulting cDNA was subjected to quantitative real-time PCR using the Power SYBR Green PCR Master Mix (Applied Biosystems) according to the manufacturers’ instructions. The primers and sequences used are listed in Supplementary Table 1.

### Western blot analysis

Cells were washed in ice-cold PBS and lysed on ice using cell lysis buffer (9803, Cell Signaling Technology) for total cellular protein. Tissues were homogenized and lysed in T-per Tissue protein extraction reagent (78510, Thermo Fisher Scientific) using a bead homogenizer for 3 min at maximum speed (Qiagen, TissueLyser II) for total tissue protein. Membrane-and-cytoplasmic protein fractions of cultured cells and tissue were obtained with the Mem-PER Plus Membrane Protein Extraction Kit (Pierce Protein Biology) for membrane cellular and tissue protein. Each lysate was supplemented with protease inhibitor cocktail (#P8340, Sigma-Aldrich), phosphatase inhibitor cocktail 2 (#P5726, Sigma) and phosphatase inhibitor cocktail 3 (#P0044, Sigma). Lysates were centrifuged at 16,000 rpm for 15 min at 4 °C, and protein concentrations were quantified using the BCA Protein Assay Kit (71285-M, Sigma). Equal amounts of lysate were mixed with NuPAGE LDS Sample Buffer (4×) (Thermo Fisher Scientific, cat. no. NP0007) and boiled for 10 min at 70 °C before being subjected to electrophoresis in NuPAGE 4–12% Bis-Tris Protein Gels (Thermo Fisher Scientific, cat. no. NP0336BOX). Proteins were resolved on a 4%–20% gradient SDS–PAGE, transferred to polyvinylidene fluoride membranes and probed with antibodies for GLUT1 (Abcam, ab115730), TXNIP (Cell Signaling, D5F3E), GLUT2 (Novus, NBP2-22218), SGLT1 (Novus, NBP2-20338), β-actin (Santa Cruz, sc-47778) and Na⁺/K⁺-ATPase (ATP1A1) (Abcam, ab76020).

### Electroporation

After a 1-week acclimatization, cells were collected at a density of 1 × 10^6^ cells and mixed with plasmid diluted in Opti-MEM reduced serum medium (Thermo Fisher Scientific). Transfection was achieved through electroporation using the NEPA21 system following the electroporation protocol.

### Histology and immunostaining

Intestinal tissues from the animal models were paraffin-embedded and sectioned. Tissue sections (4 µm thick) were cut from each block for either hematoxylin and eosin staining or immunostaining with the following antibodies: rabbit anti-GLUT1 (Abcam, ab115730) and rabbit anti-TXNIP (Abcam, ab188865).

### Flow cytometry

Cells from in vitro experiments were collected on ice in 10 mM EDTA in PBS and fixed with 4% paraformaldehyde before flow cytometric analysis. Cells were incubated with 1:100 anti-rabbit GLUT1 (Abcam, ab115730) antibody in BD Stain Buffer (BSA) (BD Biosciences) 30 min at room temperature after 20 min on ice to block nonspecific binding, followed by incubation for 30 min at room temperature with 1:200 Alexa Fluor 647 (Abcam, ab150075). Cells were rinsed twice and then resuspended in fresh buffer for analysis on a BD FACS verse II flow cytometer using BD FACS Diva software (BD Biosciences).

### Immunocytochemistry

For intestinal organoids cultured on coverslips were washed with ice-cold PBS and fixed in 4% paraformaldehyde. The organoids were then incubated with anti-GLUT1 (1:500; ab115730, Abcam) for 12 h, followed by Alexa Fluor-488-conjugated rabbit, and Flash Phalloidin Red-594 (1:100, 424203, BioLegend) for 1 h. Finally, the coverslips were mounted in Vectashield (Vector Laboratories) and examined using a confocal laser-scanning microscope (Zeiss LSM780; Carl Zeiss MicroImaging).

### RNA sequencing

The total RNA concentration was calculated using Quant-IT RiboGreen (Invitrogen, R11490). To assess the integrity of the total RNA, samples were run on TapeStation RNA screentape (Agilent, 5067-5576). Only high-quality RNA preparations with RNA integrity numbers greater than 7.0 were used for RNA library construction.

A library was independently prepared with 0.5 µg of total RNA for each sample by Illumina TruSeq Stranded Total RNA Library Prep Gold Kit (Illumina, 20020599). The first step in the workflow involves removing the rRNA in the total RNA. Following this step, the remaining mRNA is fragmented into small pieces using divalent cations under elevated temperature. The cleaved RNA fragments are copied into first-strand cDNA using SuperScript II reverse transcriptase (Invitrogen, 18064014) and random primers.

This is followed by second-strand cDNA synthesis using DNA polymerase I, RNase H and dUTP. These cDNA fragments then go through an end repair process, with the addition of a single ‘A’ base, and then ligation of the adapters. The products are then purified and enriched with PCR to create the final cDNA library.

The libraries were quantified using the KAPA Library Quantification Kit for Illumina sequencing platforms, following the qPCR Quantification Protocol Guide (KAPA BIOSYSTEMS, KK4854), and qualified using the TapeStation D1000 ScreenTape (Agilent Technologies, 5067-5582). Indexed libraries were then submitted to an Illumina NovaSeq (Illumina), and the paired-end (2 × 100 bp) sequencing was performed by Macrogen Incorporated.

### Statistical analysis

All data in the current study were represented as mean ± standard error of the mean (s.e.m.), with the numbers of experiments or mice indicated in the figure legends. Unpaired two-tailed Student’s *t*-tests were used for the comparisons between two groups. Differences between multiple groups with one variable were determined using multiple *t*-tests. Statistical analyses were performed using the GraphPad Prism 6 software. Significance was defined as ^∗^*P* < 0.05, ^∗∗^*P* < 0.01, ^∗∗∗^*P* < 0.001, ^#^*P* < 0.05 and ^##^*P* < 0.01. No method was used to determine whether the data met the assumptions of the statistical approach. A power analysis was not performed to determine the sample size.

## Results

### Metformin enhances glucose uptake and excretion in the distal intestine

**Fig. 1:**
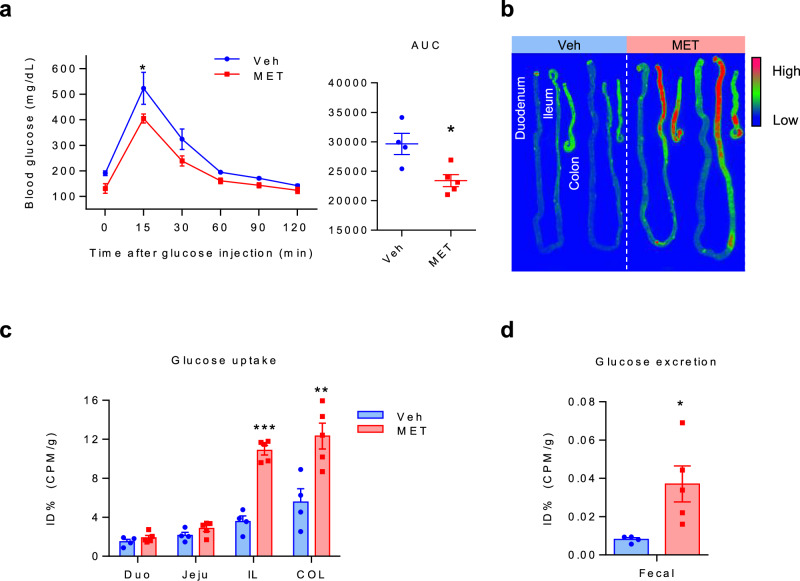
Metformin enhances blood glucose homeostasis by increasing glucotonic effect in the distal intestine and colon of C57BL/6 mice. **a** IPGTT data for vehicle (saline)- and metformin-treated mice. Metformin-treated (MET) C57BL/6 mice (*n* = 4) exhibited better glucose tolerance than vehicle-treated (Veh) mice (*n* = 4, analysis of variance multiple *t*-test for multiple-comparison correction) and corresponding area under the curve (AUC). **b** Representative images of small-intestinal and colon ^18^F-FDG) autoradiography of metformin-treated and vehicle-treated mice after PBS lavage. Areas of higher FDG accumulation are red in color. **c** A graph showing the quantitative analysis of FDG uptake in the post-washing intestine tissue autography images. Metformin-treated limbs showed higher FDG uptake in the distal intestine, such as the ileum (IL) and colon (COL), compared with that in the corresponding vehicle-treated intestine, whereas no increase was observed in the proximal intestine, such as the duodenum (DUO) and jejunum (JEJU). **d** Colon PBS washing analysis. Colon PBS washings showed a larger amount of FDG excretion in the metformin-treated mice. All data are presented as the mean ± s.e.m. Data in **a**, **c** and **d** were analyzed using two-tailed Student’s *t-*tests; *P < 0.05, **P < 0.01, ***P < 0.001.

To determine the effect of metformin on glucose regulation, we examined the effect of a single dose of metformin in C57BL/6 mice and compared the results with those obtained in vehicle-treated mice. IPGTT was performed to compare the initial responses of glucose metabolism. Metformin-treated mice exhibited enhanced blood glucose homeostasis compared with that of vehicle-treated mice (Fig. 1a). To determine the specific location of the gastrointestinal (GI) tract where metformin regulates glucose metabolism, the entire GI tract of mice was subjected to autoradiography using ^18^F-FDG as a glucose tracer. A significant increase in glucose uptake was observed in the distal intestine of metformin-treated mice compared with that in vehicle-treated mice (Fig. 1b). Analysis of ^18^F-FDG gamma counts revealed that, compared with the vehicle-treated group, the metformin-treated group exhibited higher glucose uptake in the ileum and colon than in the proximal intestine (Fig. 1c). Furthermore, FDG excretion was significantly increased in the stool of metformin-treated mice compared with that in vehicle-treated mice (Fig. 1d). These results suggest that the increased glucose uptake and excretion (glucotonic effect) in the distal intestine and colon following metformin administration indicate that metformin acts at these regions to exert its blood glucose-lowering effects.

### Metformin enhances GLUT1 expression and function in intestinal cells

**Fig. 2:**
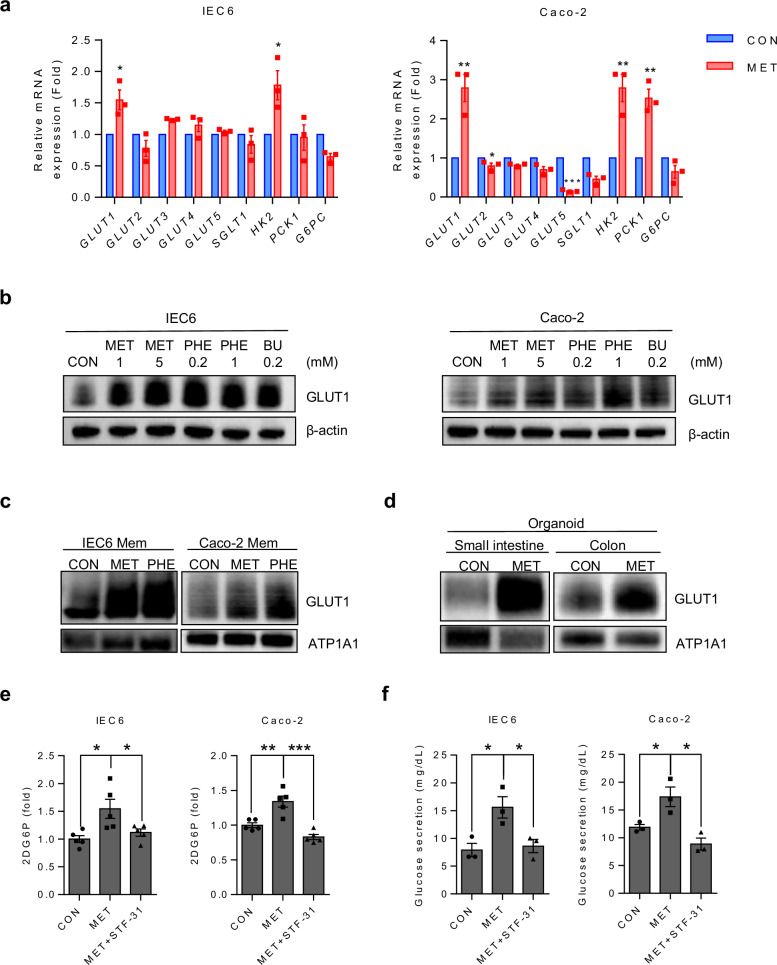
Metformin induces a glucotonic effect in small intestine and colon cell lines by upregulating GLUT1 expression and enhancing glycolysis. **a** qRT-PCR results of representative genes and corresponding fold changes in expression from metformin-treated rat small intestine (IEC6) and human colon (Caco-2) cell lines. **b**, **c** IEC6 and Caco-2 cell lines were incubated with vehicle (CON) or presence of biguanides, such as metformin (MET), phenformin (PHE) and buformin (BU) for 16 h, after 8-h starvation in serum-free medium, and later collected for immunoblotting of GLUT1: total proteins show that GLUT1 increases in IEC6 and Caco-2 cell lines after biguanide treatment (expression levels were normalized to β-actin) (**b**); membrane proteins show that GLUT1 increases in IEC6 and Caco-2 cell line plasma membranes after two representative biguanide treatment (expression levels were normalized to Na⁺/K⁺-ATPase (ATP1A1)) (**c**). **d** Immunoblots of plasma membrane lysates of organoids. ATP1A1 served as a loading control of membrane protein. **e** Increase in glucose uptake after metformin treatment in small intestine and colon cell lines. Glucose uptake detected of 2-deoxyglucose-6-phosphate (2DG6P). 2DG6P was normalized with MTS. Glucose uptake was abolished after GLUT1-specific inhibitor (STF-31) treatment in metformin-treated IEC6 and Caco-2 cell lines. **f** Increase in glucose excretion after metformin treatment in IEC6 and Caco-2 cell lines. Glucose excretion was abolished after GLUT1-specific inhibitor (STF-31) treatment in metformin-treated IEC6 and Caco-2 cell lines. All data are presented as the mean ± s.e.m. Data in **a**, **e** and **f** were analyzed using two-tailed Student’s *t*-tests; *P < 0.05, **P < 0.01, ***P < 0.001.

Previous studies have demonstrated that changes in glucose metabolism within the GI tract observed following gastric bypass surgery or metformin treatment are associated with alterations in the gene expression of glucose transporters^26,28,30–32^. To identify the specific glucose transporters that play a major role in the glucotonic effect induced by metformin, mRNA levels of multiple glucose transporters were examined using both rat ileum (IEC6) and human colon (Caco-2) cell lines. Both cell lines exhibited significantly increased expression of GLUT1 (Fig. 2a). Moreover, both cell lines also showed a significant increase in the expression of hexokinase 2 (HK2), which enables the measurement of glycolysis following glucose transport into the cell via the glucose transporter^33^. By contrast, all other transporters showed no significant change in transcript levels in IEC6 cells. However, GLUT2 and GLUT5 expression was reduced in Caco-2 cells after metformin treatment, with no significant changes observed in the other transporters (Fig. 2a). Unlike the glycolysis-related enzyme HK2, the gluconeogenesis-related enzymes phosphoenolpyruvate carboxykinase 1 (PCK1) and glucose-6-phosphatase catalytic subunit (G6PC) exhibited no consistent changes, indicating no common effect on gluconeogenesis in both IEC6 and Caco-2 cells. Notably, only PCK1 levels increased significantly in Caco-2 cells. In addition, treatment with phenformin, another biguanide, mirrored the effects of metformin, significantly increasing GLUT1 and HK2 mRNA expression in both cell lines (Supplementary Fig. 1a). Consistently, treatment with biguanides (metformin, phenformin and buformin) resulted in increased GLUT1 protein expression in both IEC6 and Caco-2 cell lines (Fig. 2b). As active GLUT1 primarily localizes to the cell membrane and facilitates glucose transport across the cell membrane^17^, we extracted membranous proteins from IEC6 and Caco-2 cells and measured GLUT1 activity after treatment with metformin or phenformin. Membranous GLUT1 levels increased following metformin and phenformin treatment in both cell lines (Fig. 2c). Although the protein levels of GLUT2 and SGLT1, intestinal glucose transporters^18,34^, showed no changes in Caco-2 cells, they increased in IEC6 cells. However, there were no significant changes in membrane proteins in either cell line after metformin treatment (Supplementary Fig. 1b,c). Next, mouse intestinal and colorectal organoids were established to investigate the effects of metformin in a setting mirroring in vivo. GLUT1 expression was increased in the plasma membranes of metformin-treated intestinal and colorectal organoids (Fig. 2d). These results suggested that metformin induces glycolysis by increasing GLUT1 expression and membrane translocation in the distal intestine. Furthermore, 2DG uptake assays revealed that metformin treatment increased glucose uptake in both intestinal cell lines (Fig. 2e). To determine the involvement of GLUT1 in mediating this effect, cells were treated with STF-31, a GLUT1-specific inhibitor^35,36^, in combination with metformin. STF-31 treatment abolished the metformin-induced increase in glucose uptake in both cell lines, confirming the role of GLUT1 in this process (Fig. 2e). Moreover, metformin treatment enhanced glucose excretion in both the IEC6 and Caco-2 cell lines (Fig. 2f). Notably, the diffusion of FITC-dextran was not observed, confirming that the excreted glucose was not the result of diffusion, but occurred via the glucose transporter (Supplementary Fig. 1d). Moreover, STF-31 treatment abolished the metformin-induced glucose excretion in both cell lines (Fig. 2f). To replicate and verify whether glucose secretion induced by metformin in organoids, we measured the amount of glucose in the medium from monolayer-seeded colon organoids. Glucose secretion was significantly increased in metformin-treated colon organoids (Supplementary Fig. 1e). These results highlight the crucial role of GLUT1 in metformin-induced increases in the intestinal glucotonic effect.

### Metformin enhances GLUT1 expression via suppressing TXNIP

**Fig. 3:**
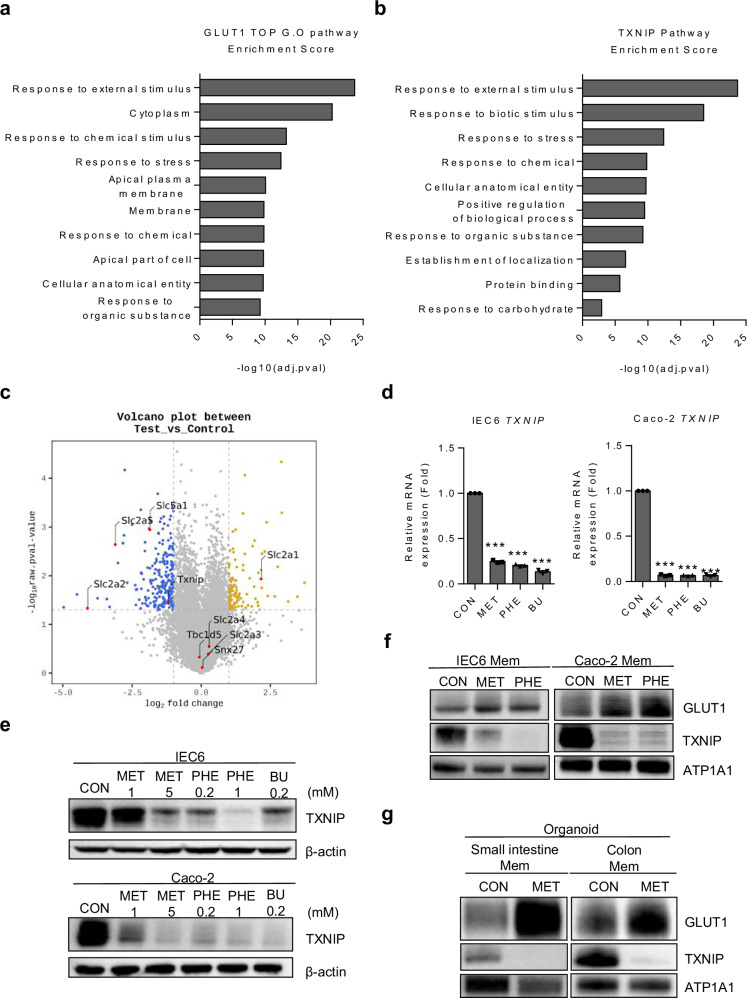
Metformin increases GLUT1 expression and its membrane localization in small intestine and colon cell lines by downregulating TXNIP. **a** Top GO pathway enrichment scores for GLUT1. **b** Enrichment scores for TXNIP-related pathways. **c** A volcano plot showing the results of RNA sequencing analysis comparing the transcriptomes of mouse ileum samples from the control group and metformin-treated group. The left side of volcano plot is p-value <0.05 and fold change ≤2; the right side of volcano plot is p-value <0.05 and fold change ≥2. **d** qRT-PCR analysis of TXNIP mRNA expression in IEC6 and Caco-2 cells treated with biguanides. **e**, **f** IEC6 and Caco-2 cell lines were incubated in the absence or presence of biguanides for 16 h, after 8-h starvation in serum-free medium, and later collected for immunoblotting of TXNIP. **e** Total proteins showing that TXNIP decreases in IEC6 and Caco-2 cell lines after biguanide treatment. Expression levels were normalized to β-actin. **f** Membrane protein analysis showing that TXNIP decreases in the plasma membranes of IEC6 and Caco-2 cells after treatment with two representative biguanides, while GLUT1 levels increase. Expression levels were normalized to ATP1A1. **g** Immunoblots of plasma membrane lysates of organoids. ATP1A1 served as a loading control of membrane protein. All data are presented as the mean ± s.e.m. Data in **d** were analyzed using two-tailed Student’s *t*-tests; ***P < 0.001.

To examine the upregulation of GLUT1 associated with metformin treatment, we compared gene expression in selected regions of the ileum showing higher FDG uptake in the metformin-treated group, with basal levels observed in the vehicle-treated group using RNA sequencing. Analysis of RNA sequencing data from ileum samples revealed significant modulation of pathways by metformin (Supplementary Fig. 2a–c). A strong correlation was observed between these pathways and the top Gene Ontology (GO) pathways, with the highest enrichment scores for GLUT1 (Fig. 3a). This observation suggests a robust association between the pathways altered by metformin and the metformin-induced increase in GLUT1 expression. To elucidate the mechanism underlying metformin-induced increase in GLUT1 expression, we comparatively analyzed the proteins implicated in RNA sequencing data that impact GLUT1 membrane trafficking^24,37–39^. Among them, GO pathways associated with TXNIP, which decreases GLUT1 expression and membrane trafficking^24,39^, exhibited high similarity with GLUT1 (Fig. 3b). Furthermore, a significant decrease in TXNIP levels was observed after metformin treatment. However, other known regulators of GLUT1 membrane trafficking, such as Sorting Nexin 27 (SNX27)^38^ and TBC1 domain family member 5 (TBC1D5)^37^, exhibited insignificant changes after metformin treatment (Fig. 3c and Supplementary Fig. 2d). Further analysis confirmed that the expression of glucose transporters other than GLUT1 was either insignificantly altered or decreased (Fig. 3c and Supplementary Fig. 2d). In addition to increasing GLUT1 expression, metformin treatment led to a consistent reduction in GLUT2, GLUT5 and SGLT1 expression across intestinal tissues (Fig. 3c), suggesting suppressed glucose and fructose absorption from the lumen to blood, potentially contributing to systemic glucose lowering^40^. To validate the metformin-induced changes in TXNIP mRNA expression, we treated IEC6 and Caco-2 cells with biguanides. Both cell lines exhibited a notable decrease in TXNIP expression after treatment with biguanides, including metformin, phenformin and buformin (Fig. 3d). However, SNX27 and TBC1D5 mRNA levels did not show a consistently significant pattern across the biguanide groups, in line with RNA sequencing data (Supplementary Fig. 2d–f). Next, we assessed the protein levels of TXNIP in IEC6 and Caco-2 cells. Treatment with metformin and other biguanides led to a dose-dependent decrease in TXNIP expression (Fig. 3e). The expression of TXNIP was decreased in both cell lines and in both mouse intestinal and colorectal organoid membrane proteins, whereas GLUT1 levels were increased by metformin administration (Fig. 3f, g). These results indicate that metformin increased GLUT1 membrane localization by decreasing TXNIP expression.

Previous research has demonstrated that metformin inhibits mitochondrial complex 1 of the electron transport chain^4,41^ and activates AMPK^5,6^; therefore, we sought to determine which of these mechanisms was associated with GLUT1 expression upregulation. IEC6 cells were subjected to treatment with metformin alone, post-metformin treatment with the AMPK inhibitor (compound C), or rotenone, a known complex 1 inhibitor^42,43^, alone. Cells co-treated with metformin and compound C exhibited upregulated GLUT1 expression and downregulated TXNIP expression compared with the control (Supplementary Fig. 3a). To further confirm whether AMPK inhibition influences metformin-induced GLUT1 upregulation, AMPK was knocked down using small interfering RNA (siRNA) as an alternative approach to pharmacological inhibition. Consistent with our previous findings, AMPK knockdown did not abolish the metformin-induced upregulation of GLUT1 expression (Supplementary Fig. 3b). These data collectively indicate that AMPK activation is not required for metformin-induced GLUT1 increase and TXNIP reduction. Rather, the metformin-mediated decrease in TXNIP expression appears to primarily involve a pathway independent of AMPK activation. Cells treated with rotenone exhibited upregulated GLUT1 expression and downregulated TXNIP expression like those treated with metformin (Supplementary Fig. 3c). In addition, to determine whether direct TXNIP suppression could mimic the effects of metformin, IEC6 cells were transfected with TXNIP-specific siRNA (siTXNIP). As expected, siTXNIP transfection decreased TXNIP expression in IEC6 cells (Supplementary Fig. 3d). These findings further support the notion that TXNIP plays a critical role in mediating metformin-induced GLUT1 upregulation.

Next, we explored the mechanism underlying metformin-induced TXNIP suppression by examining mitochondrial complex I inhibition and associated antioxidant remodeling. Because TXNIP expression decreases under conditions of reduced ROS^44^, we hypothesized that metformin lowers intracellular ROS by enhancing antioxidant defenses and NADPH availability. Analysis of our RNA sequencing data revealed a trend toward increased expression of key antioxidant genes, including GSTA1–4 and TXNRD1, after metformin treatment (Supplementary Fig. 4a). These enzymes are involved in detoxification processes and thioredoxin recycling, respectively, suggesting transcriptional activation of antioxidant systems. Notably, expression of malic enzyme 1 (ME1), a cytosolic enzyme generated from malate, was elevated (Supplementary Fig. 4a). As NADPH is crucial for antioxidant pathways, such as glutathione and thioredoxin, ME1 upregulation suggests enhanced cellular capacity for ROS. Consistently, increased ME1 and TXNRD1 expression was confirmed in intestinal tissues from metformin-treated mice, validating antioxidant remodeling in vivo (Supplementary Fig. 4b).

Moreover, genes associated with NADH consumption or conversion to NADPH, including LDHA, BADH (ALDH9A1), NNT and NADK2, were also upregulated by metformin treatment in intestinal tissues (Supplementary Fig. 4b). Considering that metformin-induced mitochondrial complex I inhibition impairs NADH oxidation and elevates NADH levels, these transcriptional changes probably represent a compensatory adaptation to restore redox homeostasis by enhancing NADPH generation. Similar results were observed in IEC6 and Caco-2 cells treated with metformin, demonstrating consistent ME1 and TXNRD1 upregulation (Supplementary Fig. 4c), supporting that metformin induces similar antioxidant remodeling in vitro. Taken together, these results support a model in which metformin suppresses TXNIP expression not through AMPK activation, but via mitochondrial complex I inhibition, leading to increased NADPH production, enhanced antioxidant gene expression and ROS reduction.

### TXNIP overexpression suppresses metformin-induced GLUT1 activation

**Fig. 4:**
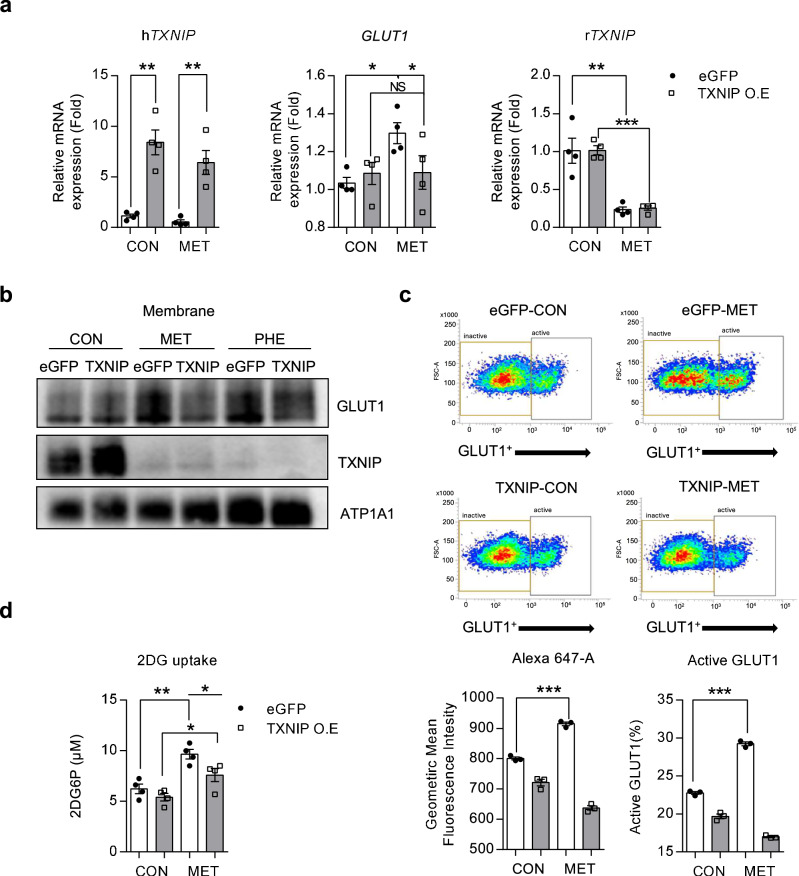
TXNIP overexpression inhibits metformin-induced upregulation of GLUT1 expression and glucose uptake in intestinal cell lines. **a** The expression of GLUT1 and TXNIP in vehicle- or metformin-treated IEC6 cells expressing EGFP or human TXNIP. **b** Membrane GLUT1 and TXNIP in vehicle- or metformin-treated EGFP or human TXNIP-overexpressing IEC6 cells. The metformin-induced increase in GLUT1 expression was blunted in TXNIP-overexpressing cells. Expression levels were normalized to ATP1A1. **c** Representative fluorescence-activated cell sorting (FACS) plots analyzing the effect of TXNIP overexpression on metformin-induced GLUT1 activation, using Alexa 647. The left graphs indicate the geometric mean fluorescence intensity, and the right graphs indicate the percentage of active GLUT1 in FACS plots. **d** Glucose uptake decreased in metformin-treated TXNIP-overexpressing IEC6 cells compared with metformin-treated EGFP-overexpressing IEC6 cells. Glucose uptake was measured by detecting 2-deoxyglucose-6-phosphate (2DG6P). 2DG6P was normalized with MTS. All data are presented as the mean ± s.e.m. Data in **a**, **c** and **d** were analyzed using two-tailed Student’s *t*-tests; *P < 0.05, **P < 0.01, ***P < 0.001. NS, nonsignificant.

We next elucidated the effect of differential TXNIP expression on GLUT1 expression in IEC6 cells overexpressing TXNIP. In these cells, the metformin-induced increase in GLUT1 expression was abolished (Fig. 4a). Furthermore, expression of membranous GLUT1 did not increase in these cells, even after metformin treatment (Fig. 4b). Fluorescence-activated cell sorting analysis revealed that the ratio of active GLUT1, which was significantly increased by metformin, was unchanged in cells overexpressing TXNIP (Fig. 4c). Furthermore, metformin-induced 2-DG uptake decreased in cells overexpressing TXNIP compared with that in nonoverexpressing cells (Fig. 4d). These findings support the hypothesis that TXNIP overexpression inhibits metformin-induced upregulation of GLUT1 expression in enterocytes, thereby influencing glucose uptake.

### Overexpression of intestinal TXNIP abolishes the glucotonic effect of metformin

**Fig. 5:**
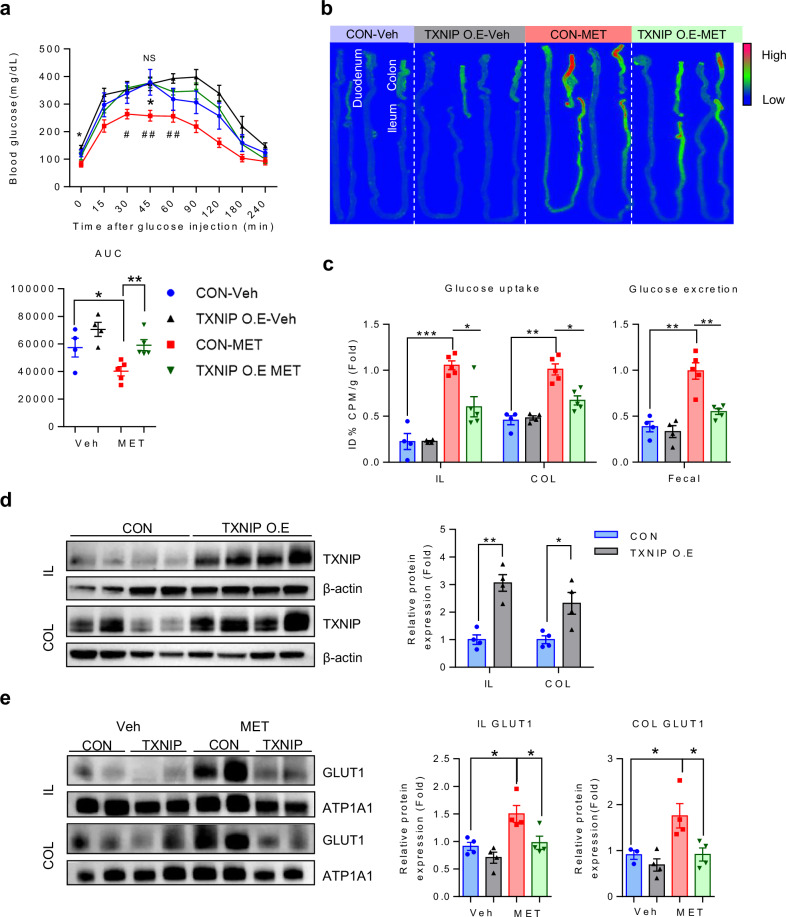
TXNIP overexpression in diabetic mice abolishes the blood glucose-lowering effect of metformin by reducing its enhancement of intestinal GLUT1 expression and glucotonic effect. **a** IPGTT data for vehicle- and metformin-treated transgenic mice. Metformin-treated nontransgenic C57BL/6 mice (*n* = 5) exhibited better glucose tolerance than both vehicle-treated mice (*n* = 4) and metformin-treated mice overexpressing TXNIP (*n* = 5, analysis of variance multiple *t*-test for multiple-comparison correction; *P < 0.05 was used to compare CON-NT and CON-MET, ^#^*P* < 0.05, and ^##^*P* < 0.01 was used to compare CON-MET and TXMIP-MET) and corresponding AUCs. **b** Representative images of small-intestinal and colon FDG autoradiography of metformin-treated and vehicle-treated mice after PBS lavage. Areas of higher FDG accumulation are red in color. **c** The right graph shows the quantitative analysis of FDG uptake in post-washing intestinal tissue autoradiography images. Metformin-treated mice exhibited higher FDG uptake in the ileum and colon compared with both vehicle-treated mice and metformin-treated mice with TXNIP overexpression. The left graph shows the analysis of PBS washings from the colon. PBS washings from metformin-treated mice contained significantly more FDG than those from vehicle-treated mice or metformin-treated mice overexpressing TXNIP, indicating greater FDG excretion. **d** Representative images of the total protein levels of TXNIP demonstrate a significant overexpression of TXNIP in the group compared with the control group, indicating robust TXNIP upregulation in the ileum and colon. The graph shows a significant increase in TXNIP expression between control and TXNIP-overexpressing groups, providing evidence of robust TXNIP upregulation. **e** Representative images of the expression analysis of GLUT1 in membrane proteins revealing a significant increase in GLUT1 expression in the mouse intestine after metformin treatment, whereas a decrease was observed in mice overexpressing TXNIP, in both the ileum and colon. All data are presented as the mean ± s.e.m. Data in **a** and **c**–**e** were analyzed using two-tailed Student’s *t*-tests; *P < 0.05, **P < 0.01, ***P < 0.001. NS, nonsignificant.

TXNIP overexpression was induced to examine the reduction in metformin efficacy and to assess the replicability of TXNIP regulation in diabetes animal models. TXNIP overexpression was induced in STZ-induced diabetic mice. The metformin dose administered to the mice in this experiment was determined to be at a therapeutically appropriate level when translated to the treatment of patients with diabetes^45^. Notably, the blood glucose-lowering effect of metformin was abolished in the diabetic mice overexpressing TXNIP (Fig. 5a). A reduction in metformin-induced enhancement of the glucotonic effect in the intestine was also abolished in TXNIP-overexpressing diabetic mice (Fig. 5b, c). To corroborate these findings, we assessed protein expression in the ileum and colon and observed TXNIP overexpression in both tissues. TXNIP protein expression increased quantitatively in the ileum and colon tissue (Fig. 5d). Conversely, no significant changes in TXNIP protein expression were noted in other organs, including the liver, kidney, heart, brain, muscle, white adipose tissue and pancreas (Supplementary Fig. 5a), indicating an association between increased TXNIP levels in the distal intestine and metformin-induced blood glucose levels (Fig. 5a). Subsequent examination of membranous GLUT1 expression in both tissues revealed that TXNIP overexpression led to a significant decrease of GLUT1 expression in the ileum and colon after metformin treatment, where metformin increased GLUT1 expression (Fig. 5e). These results suggest an association between metformin-induced enhancement of intestinal glucose uptake and excretion, and TXNIP-mediated regulation of intestinal GLUT1 in diabetic mice.

### Intestinal GLUT1 activation is pivotal for metformin-induced glucose homeostasis

**Fig. 6:**
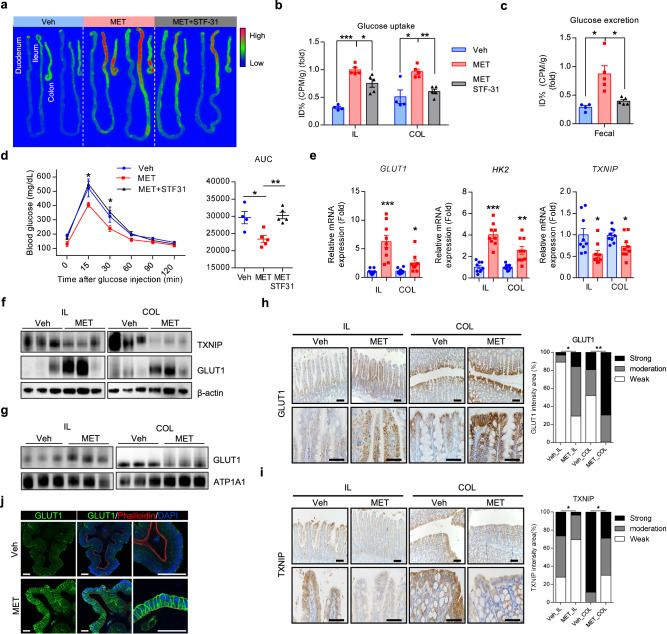
Inhibition of GLUT1 expression abolishes the metformin-induced increase in glucose homeostasis and glucotonic effect in the distal intestine. **a** Post-washing FDG autoradiography of intestinal tissues from vehicle-treated mice, metformin-treated moce and metformin-treated mice receiving intraperitoneal injection of STF-31 (a GLUT1-specific inhibitor). **b** FDG autoradiography of the relative radiotracer amount in the ileum (IL) and colon (COL) after washing. The STF-31-injected ileum and colon show significantly lower FDG uptake than the metformin-treated ileum and colon. The vehicle-treated ileum and colon were used for comparison. **c** Colon PBS washing analysis. PBS washings from the colon of STF-31-injected mice showed lower FDG excretion compared with those from metformin-treated mice. The vehicle-treated ileum and colon were used for comparison. **d** IPGTT data comparing metformin-treated mice, metformin-treated mice with GLUT1 inhibitor (STF-31) intraperitoneal injection and vehicle-treated mice. The metformin group showed significant improvement in glucose tolerance compared with the vehicle group. This improvement in glucose tolerance was significantly reduced in the metformin with STF-31 injection group compared with the metformin group (*n* = 5, analysis of variance multiple *t*-test for multiple-comparison correction) and corresponding area under the curves (AUCs). **e** The expression of GLUT1, HK2 and TXNIP in the ileum and colon from vehicle- and metformin-treated mice. **f** An immunoblot showing the increased GLUT1 and decreased TXNIP from ileum and colon samples from metformin-treated mice (*n* = 3 mice per group). Expression levels were normalized to β-actin. **g** Membrane GLUT1 expression in ileum and colon from vehicle- and metformin-treated mice (*n* = 3 mice per group). Expression levels were normalized to ATP1A1. **h**, **i** Representative images of GLUT1 (**h**) and TXNIP (**i**) immunostaining of the ileum and colon from vehicle-treated and metformin-treated C57BL/6 mice. Scale bars, 100 μm (left images) and 50 μm (right images). Statistical comparisons were performed on the percentage of strongly 3,3′-Diaminobenzidine (DAB)-stained area between vehicle- and metformin-treated groups. **j**, Representative fluorescence images of GLUT1 after vehicle and metformin treatment in human intestinal organoids. Red signals represent phalloidin 4 staining, indicating F-actin fibers. Blue signals represent DAPI staining, indicating nuclei location. Green signals represent GLUT1 expression. Scale bars, 50 μm. All data are presented as the mean ± s.e.m. Data in **b**–**e** were analyzed using two-tailed Student’s *t*-tests; *P < 0.05, **P < 0.01, ***P < 0.001.

Next, we investigated the potential role of GLUT1 inhibition in mediating the glucotonic effects of metformin in vivo. For this purpose, STF-31, a specific inhibitor of GLUT1, was utilized^35,36^. Glucose uptake was significantly reduced in the distal intestine of mice co-treated with metformin and STF-31 compared with that in metformin-only-treated mice (Fig. 6a, b). Similarly, glucose excretion was also reduced in mice co-treated with metformin and STF-31 compared with that in metformin-only-treated mice (Fig. 6c). The enhanced glucose homeostasis, which is associated with the intestinal glucotonic effect, was also abolished after inhibition of GLUT1 expression in metformin-treated mice (Fig. 6d). However, these mice did not exhibit any significant changes in body weight in response to metformin treatment or co-treatment with metformin and STF-31 (Supplementary Fig. 6a). These results suggest that the metformin-induced increase in glucose homeostasis was abolished by inhibition of GLUT1 expression, particularly in the distal intestine. Furthermore, mRNA and protein expression levels were assessed in the ileum and colon, where a significant increase in the glucotonic effect was observed after metformin treatment. Both the ileum and colon exhibited significantly increased mRNA levels of GLUT1 and HK2 and decreased levels of TXNIP (Fig. 6e). Conversely, no significant increase was observed in the expression of other glucose transporters (Supplementary Fig. 6b). Instead, reduced GLUT2 and GLUT5 expression was observed (Fig. 3c and Supplementary Fig. 2d). Moreover, no significant alterations were detected in the expression of gluconeogenesis-related enzymes PCK1 and G6PC (Supplementary Fig. 6b). The protein expression of GLUT1 was increased, whereas that of TXNIP decreased in the ileum and colon tissues treated with metformin (Fig. 6f and Supplementary Fig. 6c). In addition, membranous protein expression of GLUT1 in both the ileum and colon increased in metformin-treated mice (Fig. 6g). Subsequently, immunochemistry analysis was performed focusing on the ileum and colon, which exhibited metformin-induced glucose uptake and excretion, to elucidate the expression patterns of GLUT1 and TXNIP. In each of the four magnified figures, GLUT1 staining intensity was categorized into three levels—strong, moderate and weak—and the distribution of these expression levels was compared across groups. Immunohistochemistry images revealed a clear increase in GLUT1 expression in both the ileum and colon of metformin-treated mice (Fig. 6h). Quantitative analysis of staining intensity revealed a significant increase in moderate-to-strong GLUT1 expression after metformin treatment (Fig. 6h). The same analysis for TXNIP showed the opposite pattern, characterized by a significant increase in weak staining intensity in both the ileum and colon of metformin-treated mice, indicating downregulation of TXNIP (Fig. 6i). Notably, in vehicle-treated mice, TXNIP expression was relatively high at the villus tips of the ileum, whereas GLUT1 expression was comparatively lower in these regions (Fig. 6h, i). In addition, immunohistochemistry analysis revealed no significant differences in GLUT1 and TXNIP protein levels in the ileum and colon of mice co-treated with metformin and STF-31 (Supplementary Fig. 6d). Our findings suggested that metformin increased glucose homeostasis by increasing GLUT1 expression and reducing TXNIP levels.

Finally, to confirm whether metformin also increased GLUT1 expression in the human intestine, human intestinal organoids were treated with metformin, and GLUT1 expression was compared. In human intestinal organoids, F-actin fibers are predominantly located on the apical side. GLUT1 expression was increased on both the apical and basolateral sides of metformin-treated human intestinal organoids (Fig. 6j). These results demonstrate that metformin suppresses TXNIP expression through mitochondrial complex 1 inhibition, leading to the upregulation of GLUT1 expression and membrane localization in the ileum and colon, where it contributes to the glucotonic effect. Moreover, metformin-induced intestinal glucotonic effects play a crucial role in the regulation of glucose homeostasis.

## Discussion

This study demonstrated that metformin exerts its glucose-lowering effects primarily by modulating intestinal glucose handling through increased glucose uptake and excretion in the distal intestine, specifically the ileum and colon. These effects result from enhanced expression and membrane trafficking of GLUT1, mediated via TXNIP downregulation due to mitochondrial complex I inhibition. Consistent with this mechanism, metformin treatment reduced blood glucose in both diabetic and nondiabetic mouse models, accompanied by increased GLUT1 and decreased TXNIP expression, compared with other glucose transporters. These findings were further validated in intestinal cell lines derived from rats and humans, as well as mouse and human intestinal organoids.

Metformin is known to enhance glucose homeostasis partly by increasing incretin hormone secretion, particularly GLP-1^46,47^. To explore alternative mechanisms—especially its reported effect on intestinal glucose uptake—we aimed to minimize the confounding influence of incretin signaling. We therefore compared metformin’s effects via both oral glucose tolerance test (OGTT) and IPGTT, as the latter is known to elicit a weaker incretin response^48,49^. As shown in Supplementary Fig. 7a, metformin lowered blood glucose in both tests; however, the reduction occurred earlier in OGTT and was delayed in IPGTT, consistent with prior studies. Insulin levels were higher in OGTT than in IPGTT, while GLP-1 levels remained relatively similar across groups, with only a modest increase in the OGTT-MET (Metformin treated mice) group (Supplementary Fig. 7b). Based on these findings, we selected IPGTT as our primary method to evaluate metformin’s incretin-independent actions.

Previous investigations using FDG imaging have associated increased intestinal glucose uptake with improved glucose regulation after bariatric surgery and metformin treatment^26,28^. Although the AMPK and activating transcription factor 4 (ATF4) pathways have previously been implicated in GLUT1 regulation, our findings indicate that metformin-induced GLUT1 upregulation occurs independently of both. AMPK inhibition, either pharmacologically using compound C or through siRNA-mediated knockdown, did not block the increase in GLUT1 expression after metformin treatment (Supplementary Fig. 3a,b), supporting an AMPK-independent mechanism. Regarding ATF4, previous studies have reported that metformin increases its expression in vitro^26,50^. Consistent with these reports, we observed elevated ATF4 levels in intestinal cell lines after metformin treatment (Supplementary Fig. 8a). However, this effect was not reproduced in vivo: in the ileum and colon of metformin-treated mice, GLUT1 expression was markedly increased, whereas ATF4 levels remained unchanged (Supplementary Fig. 8b, GSE271623). These findings indicate that ATF4 does not contribute to GLUT1 regulation in intestinal tissues. Instead, our data (Fig. 3c and Supplementary Fig. 4a,b) support a mechanism in which mitochondrial complex I inhibition promotes antioxidant gene expression and NADPH production, leading to TXNIP suppression and subsequent GLUT1 upregulation.

Among various glucose transporters, GLUT1 was uniquely and consistently upregulated by metformin, and its expression remained elevated even under AMPK inhibition (Supplementary Fig. 3a,b). Similarly, treatment with either rotenone or siTXNIP—targeting mitochondrial complex I or TXNIP, respectively—recapitulated the metformin-induced increase in GLUT1 (Supplementary Fig. 3c,d). These results suggest that metformin promotes GLUT1 expression through TXNIP suppression, independently of AMPK, and probably via mitochondrial complex I inhibition. Supporting this mechanism, metformin also increased the expression of antioxidant-related enzymes in intestinal tissues and cells (Supplementary Fig. 4a–c), including ME1, which generates NADPH to support antioxidant activity. Genes involved in the conversion or consumption of excess NADH, which may accumulate due to complex I inhibition, were similarly upregulated (Supplementary Fig. 4b). These findings suggest that metformin suppresses TXNIP expression through a redox-based, AMPK-independent mechanism, thereby promoting GLUT1 upregulation. Consistent with this mechanism, inhibition of GLUT1 abolished the glucose-lowering effect of metformin in mice (Fig. 6d), highlighting the critical role of GLUT1 in mediating the metabolic actions of metformin.

In addition to increased GLUT1 expression, our data consistently showed reduced expression of GLUT2 and GLUT5 after metformin treatment (Figs. 2a and 3c and Supplementary Fig. 6b). SGLT1 was also downregulated under metformin treatment (Fig. 3c). GLUT2 is localized to the basolateral membrane of enterocytes and facilitates glucose transfer from the intestinal lumen into the bloodstream, thereby contributing to elevated blood glucose levels^18,40,51^. Similarly, SGLT1 is expressed on the apical membrane and mediates active glucose uptake from the lumen into enterocytes^18,40^. The downregulation of both transporters may thus contribute to reduced systemic glucose levels. Supporting this interpretation, a previous clinical study reported that individuals carrying SNPs associated with reduced GLUT2 expression exhibit greater responsiveness to metformin, further suggesting that decreased intestinal glucose absorption mediated by reduced expression of glucose transporters such as GLUT2 may play an important role in enhancing overall glucose homeostasis after metformin administration^52^. The decrease in GLUT5, a fructose transporter, may reflect a compensatory response to increased GLUT1-mediated glucose efflux or represent an independent regulatory effect^53^. Moreover, the concurrent increase in HK2 expression, without a corresponding rise in gluconeogenic enzymes (G6PC and PCK1), suggests preferential glycolytic rather than gluconeogenic metabolism in the intestine after metformin administration. Interestingly, we observed a modest increase in PCK1 expression in Caco-2 cells and the colon, although not statistically significant in the latter, implying that gluconeogenic responses may vary between the ileum and colon.

While concerns could arise regarding potential oncogenic effects due to increased GLUT1 expression, existing evidence strongly supports metformin’s anticancer properties^54–60^. Indeed, increased GLUT1 expression localized to intestinal epithelial membranes facilitates luminal glucose excretion, potentially reducing intracellular glucose accumulation and mitigating the Warburg effect, a hallmark of tumor metabolism. Thus, the enhanced GLUT1 expression induced by metformin in our model probably complements its well-established antitumor actions.

Despite its robust findings, this study has limitations. First, we did not include direct analysis of human intestinal tissues from metformin-treated individuals; however, consistent results obtained from human intestinal cell lines and organoids strengthen the translational relevance of our findings. In addition, while we effectively achieved intestine-specific TXNIP overexpression via plasmid-mediated delivery, long-term effects were not evaluated. Nevertheless, previous long-term studies of TXNIP overexpression indicate persistent glucose dysregulation without affecting body weight^61^, supporting the physiological significance of TXNIP suppression for glucose homeostasis. Although we used a mild STZ-induced diabetic mouse model that mimics certain aspects of type 2 diabetes, the inclusion of additional insulin-resistant or diet-induced models in future studies could enhance generalizability. Nonetheless, despite these limitations, our study offers significant insights into the direct impact of metformin on intestinal glucose metabolism, highlighting the role of TXNIP downregulation and GLUT1 upregulation as a key mechanism of its glucose-lowering effect. The consistent findings across various species and models strengthen the translational potential of this mechanism, providing a foundation for future research aimed at refining therapeutic strategies and highlighting the potential of modulating GLUT1 expression in various disease contexts.

In conclusion, our study uncovers a novel intestinal mechanism underlying metformin’s glucose-lowering action. By inhibiting mitochondrial complex I and suppressing TXNIP, metformin promotes GLUT1 expression and trafficking to intestinal epithelial membranes, increasing glucose excretion and uptake in the distal intestine. These findings highlight the therapeutic potential of targeting the intestinal TXNIP–GLUT1 axis and offer novel avenues for improving glucose homeostasis beyond conventional antidiabetic strategies.

## Supplementary information

Supplementary Information

## Data Availability

All data are available in the Article or its Supplementary Information and can be made available by request to the corresponding author. RNA sequencing data were deposited at NIH Gene Expression Omnibus (GEO) and are publicly available as of the date of publication at accession number GEO: GSE271623.
