# Author Correction: Novel mechanism whereby metformin improves glucose homeostasis: TXNIP–GLUT1 axis modulation enhances intestinal glucotonic effects

**DOI:** 10.1038/s12276-025-01541-x

**Published:** 2025-09-05

**Authors:** Chan Woo Kang, Jung Ho Nam, Ju Hun Oh, Eun Kyung Wang, Soohyun Lee, Hye Ju Shin, Ye Bin Kim, Eun Jig Lee, Byung Kook Lim, Sungsoon Fang, Arthur Cho, Cheol Ryong Ku

**Affiliations:** 1 Endocrinology, Institute of Endocrine Research, Department of Internal Medicine, Yonsei University College of Medicine, Seoul, Republic of Korea; 2 Department of Internal Medicine, Graduate School of Medical Science, Brain Korea 21 Project, Yonsei University College of Medicine, Seoul, Republic of Korea; 3 Neurobiology Section, Division of Biological Sciences, University of California, San Diego, La Jolla, CA USA; 4 Department of Biomedical Sciences, Gangnam Severance Hospital, Yonsei University College of Medicine, Seoul, Republic of Korea; 5 Department of Nuclear Medicine, Yonsei University College of Medicine, Seoul, Republic of Korea

**Keywords:** Endocrine system and metabolic diseases, Membrane trafficking, Mechanisms of disease

Correction to: *Experimental & Molecular Medicine* 10.1038/s12276-025-01518-w; article published online 6 August 2025

In this article the author’s name Sungsoon Fang was incorrectly written as Sung Soon Fang. The original article has been corrected.

