# Supplementary material for: Novel mechanism whereby metformin improves glucose homeostasis: TXNIP–GLUT1 axis modulation enhances intestinal glucotonic effects
Source: Exp Mol Med. 2025 Aug 6;57(8):1775–88. doi: 10.1038/s12276-025-01518-w (PMC12411643; doi:10.1038/s12276-025-01518-w)
Supplement: Supplementary file 1 — Supplementary Information [file 12276_2025_1518_MOESM1_ESM.pdf]

Supplementary Data

Novel Mechanism whereby Metformin Improves Glucose Homeostasis: TXNIP-GLUT1 Axis Modulation Enhances Intestinal Glucotonic Effects

Chan Woo Kang[1,6], Jung Ho Nam[2,6], Ju Hun Oh[1], Eun Kyung Wang[1], Soo Hyun Lee[1], Hye Ju Shin[1], Ye Bin Kim[2,] Eun Jig Lee[1], Byung Kook Lim[3], Sung Soon Fang[2,4], Arthur Cho[5], and Cheol Ryong Ku[1]

[1]Endocrinology, Institute of Endocrine Research, Department of Internal Medicine, Yonsei University College of Medicine, Seoul, 03722, Republic of Korea. [2]Brain Korea 21 PLUS Project for Medical Science, Yonsei University, Seoul, 03722, Republic of Korea. [3]Neurobiology Section, Division of Biological Sciences, University of California, San Diego, La Jolla, 92037, CA, USA. [4]Department of Biomedical Sciences, Gangnam Severance Hospital, Yonsei University College of Medicine, Seoul 03722, Republic of Korea. [5]Department of Nuclear Medicine, Yonsei University College of Medicine, Seoul, 03722, Republic of Korea. [6]These authors contributed equally to this work.

Corresponding author: Cheol Ryong Ku, Endocrinology, Institute of Endocrine Research, Department of Internal Medicine, Yonsei University College of Medicine, Seoul, 03722, Republic of Korea. E-mail: CR079@yuhs.ac. Phone: +82-2-2228-0833

Supplementary Data for Supplementary Fig. 1–8, Supplementary Table 1, Methods and References

Supplementary Figure

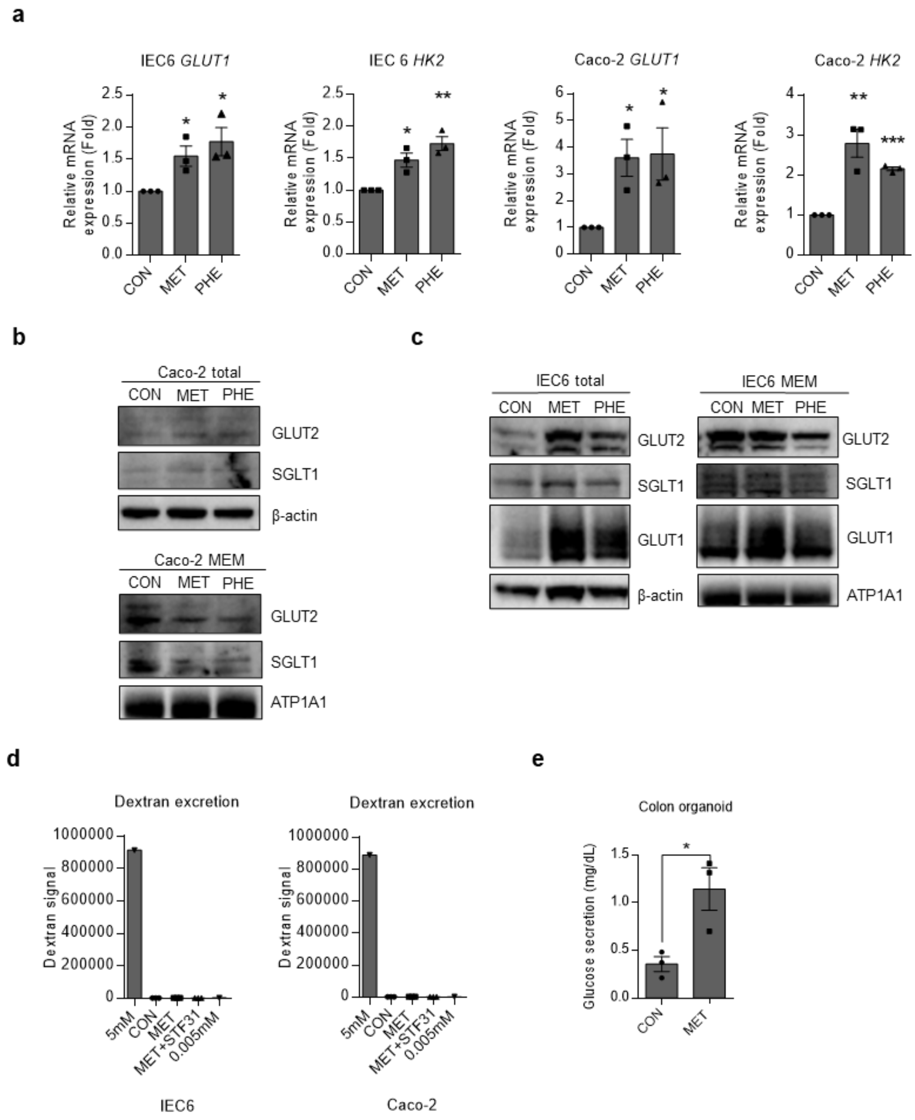

Supplementary Fig. 1. Metformin and Phenformin do not increase protein level of GLUT2 and SGLT1 and Metformin-induced glucose excretion increase was not result of diffusion. **a** qRT-PCR result of glucose transporter 1(GLUT1) and glycolysis (HK2) mRNA in biguanides (metformin, phenformin)-treated IEC6 and Caco-2. **b-c** IEC6 and Caco-2 cell lines were incubated in the absence or presence of biguanides for 16 hours, after 8 hours starvation in serum free media, and later collected for immunoblotting of GLUT2 and SGLT1. **(b)** Total and membrane protein of Caco-2. **(c)** Total and membrane protein of IEC6. **d** Dextran signal in down well of transwell compared to standard signal 5mM (input amount of upper wells) and 0.005mM. Dextran signal of media used in Fig. 2E. **e** Glucose excretion increased in metformin treated monolayer colon organoid. Quantifying based on the protein amount in each well**.** Data are presented as the mean ± SEM. Data in b was analyzed using two-tailed Student's t-tests; *P < 0.05

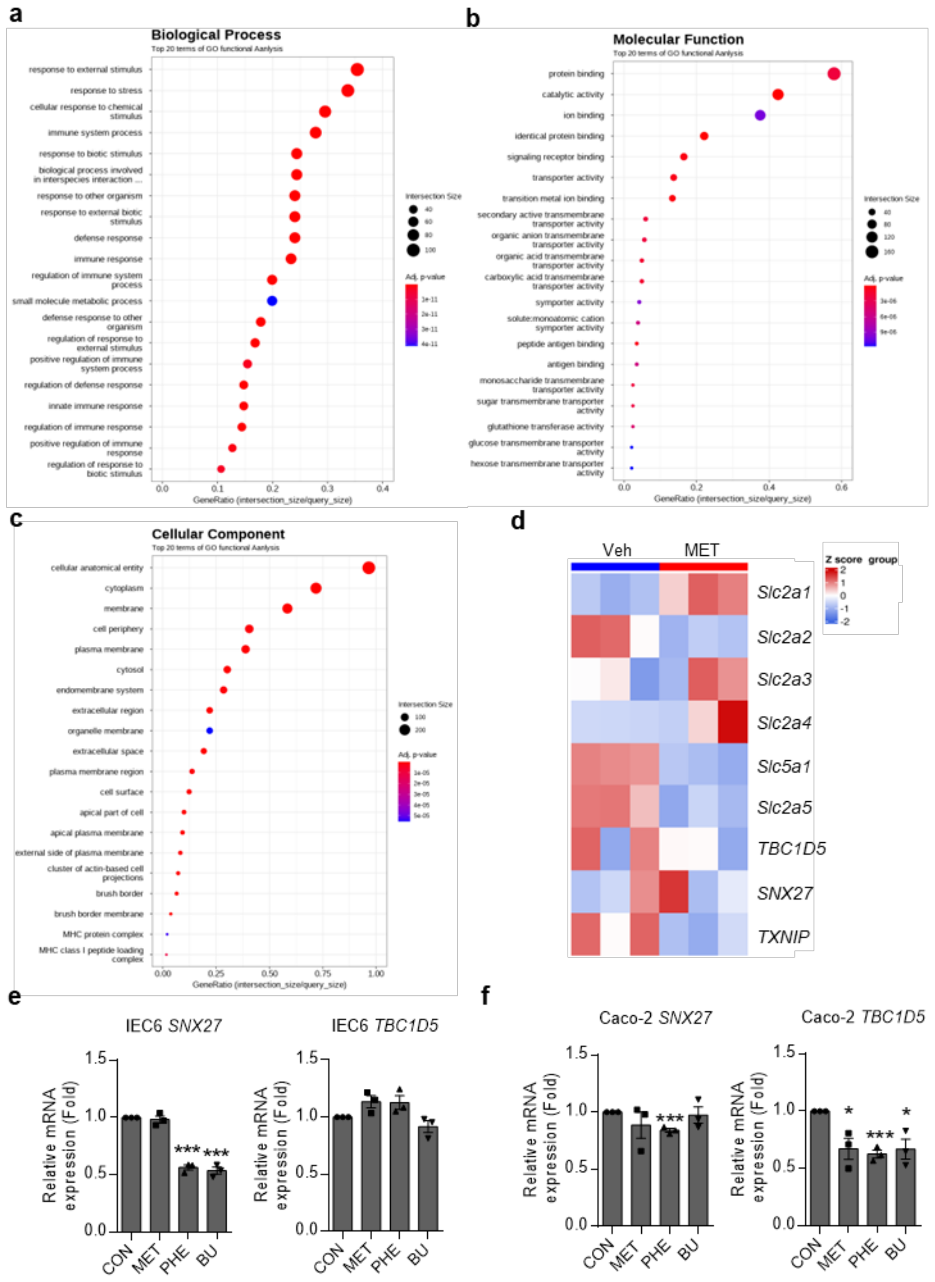

Supplementary Fig. 2. RNA changes after metformin treated in mouse ileum and IEC6 and Caco-2 cell line. **a-c** TOP 20 terms of GO functional Analysis of **(a)** Biological Process **(b)** Molecular Function **(c)** Cellular Component after metformin gavaged mice ileum. **d** Heatmap of various glucose transporters and protein related to GLUT1 membrane localization. **e-f** qRT-PCR result of TXNIP, SNX27 and TNC1D5 mRNA with treated biguanides to **e** IEC6 and **f** Caco-2. All data are presented as the mean ± SEM. Data in e and f was analyzed using two-tailed Student's t-tests; *P < 0.05, ***P < 0.001.

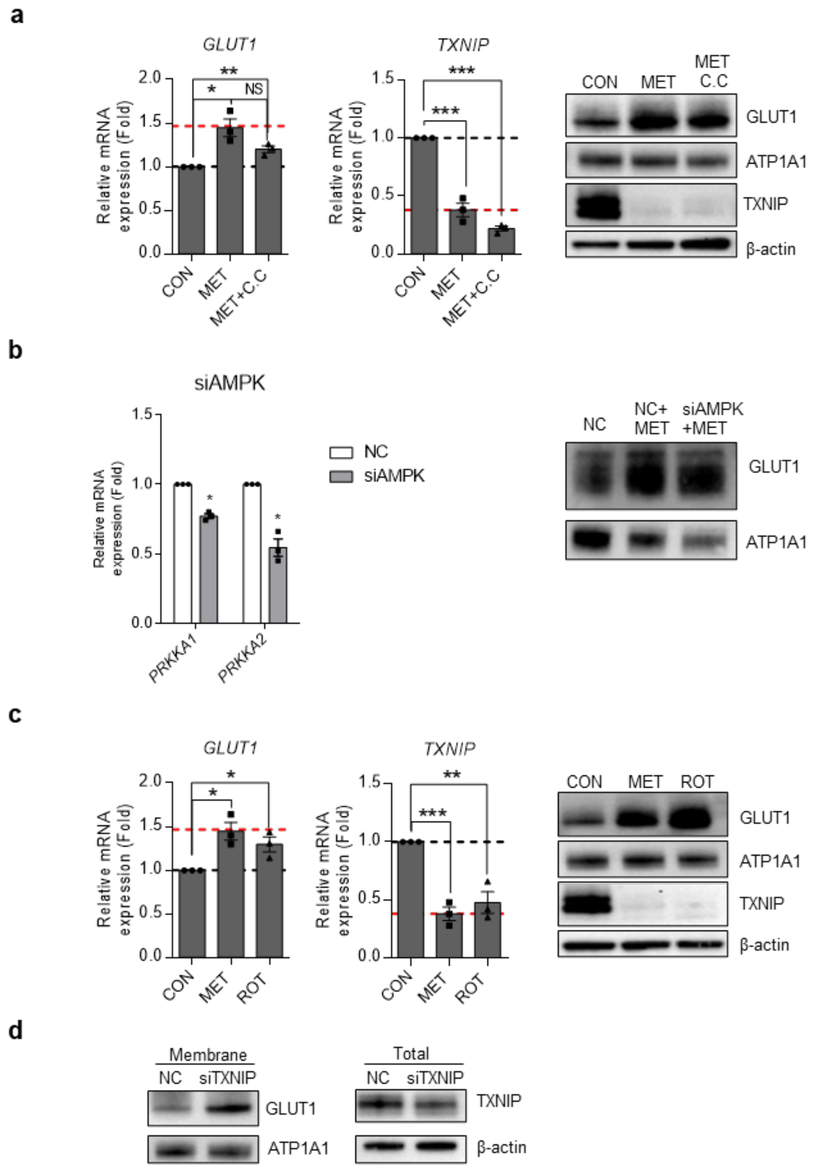

Supplementary Fig. 3. Metformin increased GLUT1 expression and translocation related to not AMPK but the inhibition of complex 1. a qRT-PCR and western result of GLUT1 and TXNIP after treating AMPK inhibitor. Black dot line indicates level of control and red dot line indicates level of metformin group. Metformin and cotreated with metformin and AMPK inhibitor (compound c). **b** qRT-PCR and western result after transfect with siAMPK **c** qRT-PCR and western result of GLUT1 and TXNIP after treating complex1 inhibitor. Black dot line indicates level of control and red dot line indicates level of metformin group. Metformin and complex1 inhibitor (Rotenon). **d** Western result after transfect with siTXNIP. All data are presented as the mean ± SEM. Data in a and b were analyzed using two-tailed Student's t-tests; *P < 0.05, **P < 0.01, ***P < 0.001.

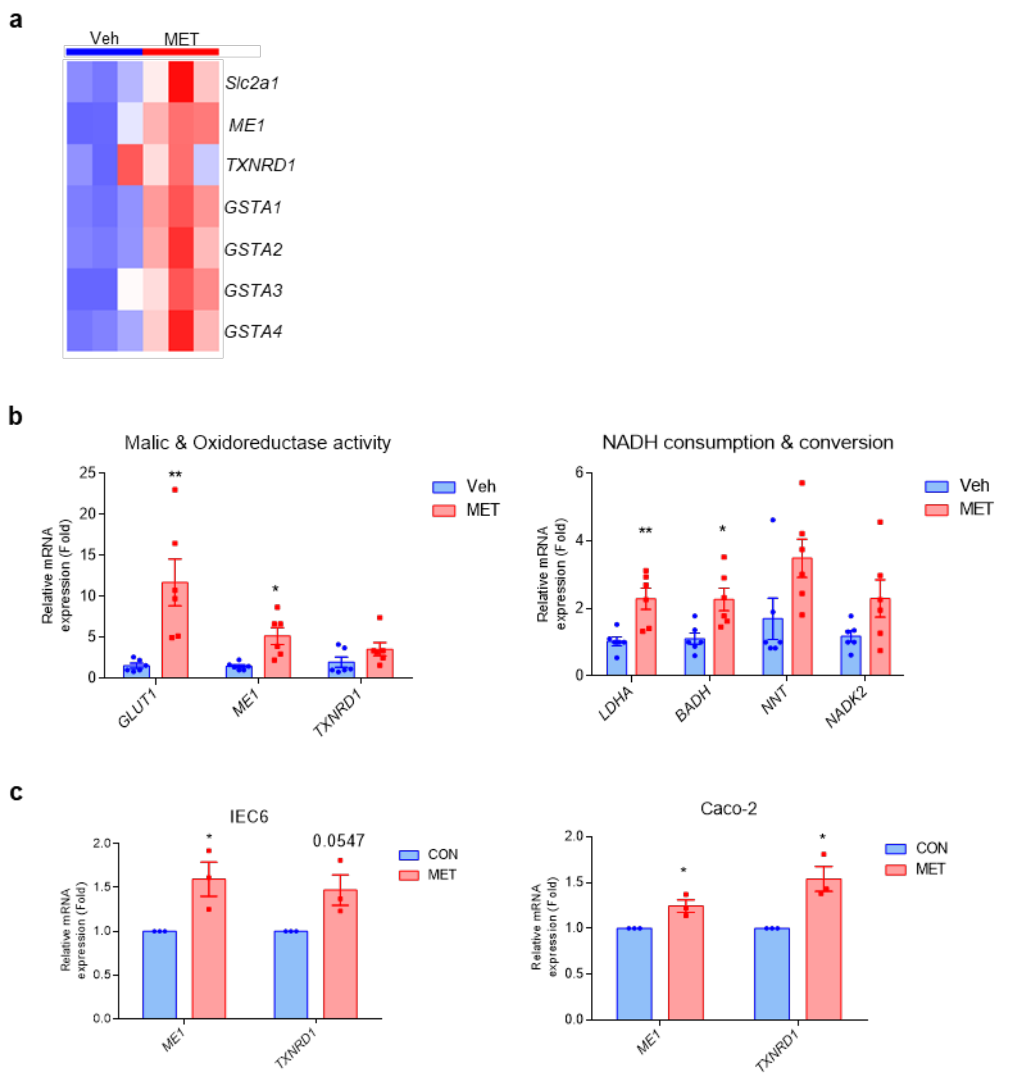

Supplementary Fig. 4. Metformin suppresses TXNIP expression via ROS reduction through antioxidant remodeling a Heatmap of various key antioxidant enzymes and NADPH production enzyme. **b** qRT-PCR result of NADPH production, antioxidant enzyme and NADH comsumption enzymes in intestinal tissues. **c** qRT-PCR result of NADPH production, antioxidant enzyme in IEC6 and Caco-2. All data are presented as the mean ± SEM. Data in a and b were analyzed using two-tailed Student's t-tests; *P < 0.05, **P < 0.01.

a

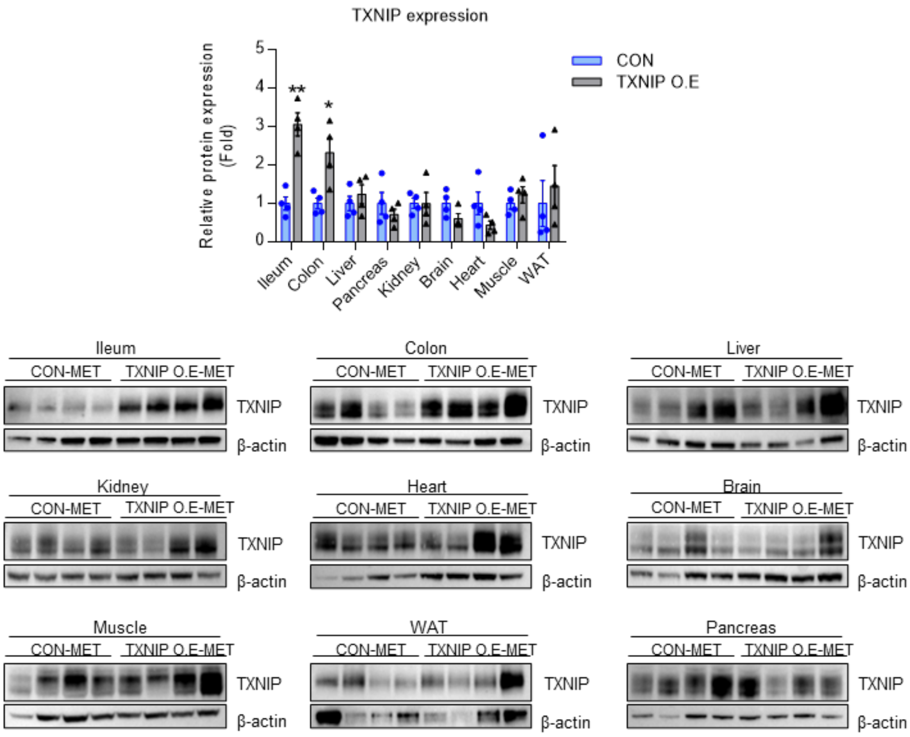

Supplementary Fig. 5. Intestinal TXNIP inhibits metformin-induced glucose homeostasis.

a Result of TXNIP expression in various organ compare with metformin-treated mice and metformin treated in TXNIP overexpressed transgenic mice. Graph is sum of western data quantifying based on the western data with ImageJ**.** Data are presented as the mean ± SEM. Data a was analyzed using two-tailed Student's t-tests; *P < 0.05, **P < 0.01.

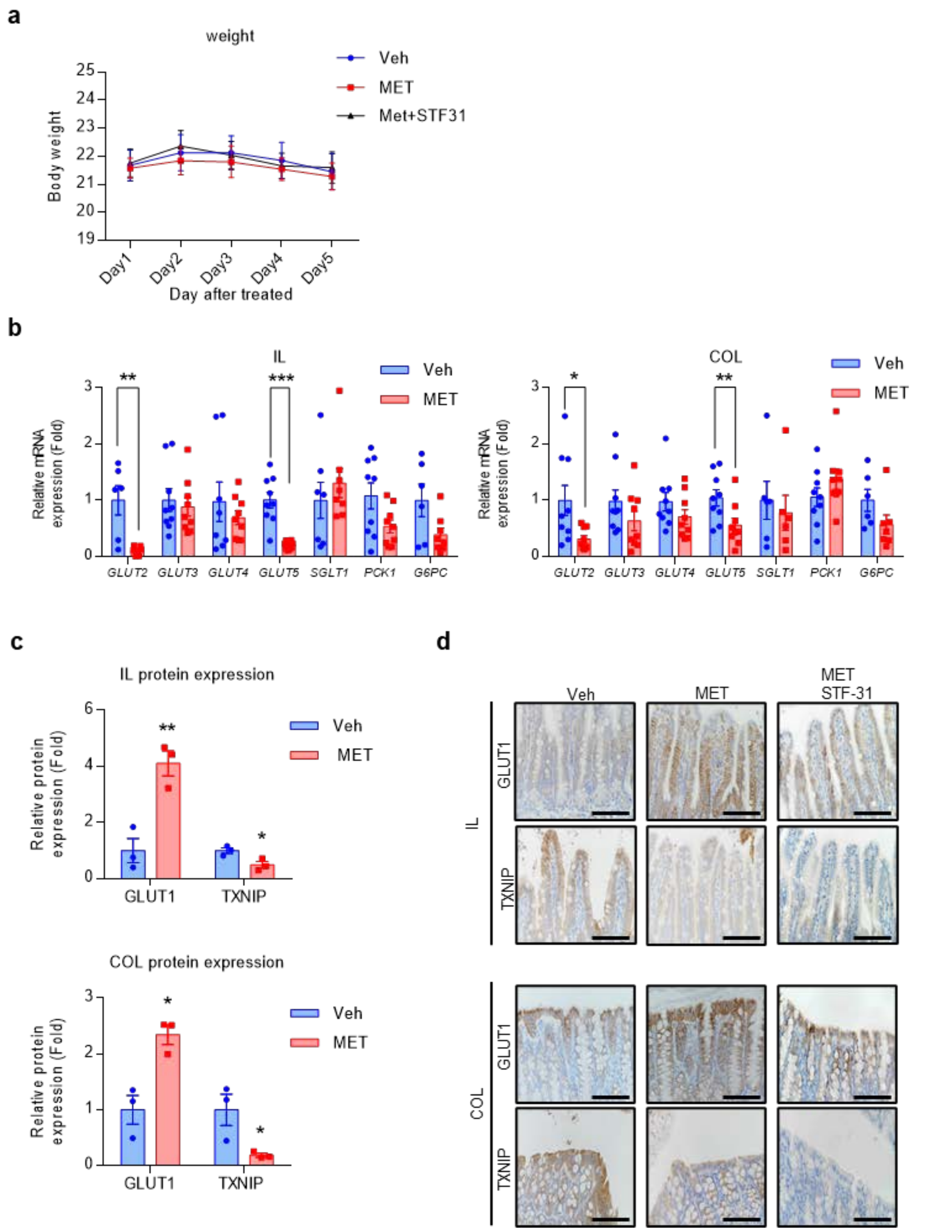

Supplementary Fig. 6. Metformin increases glucose homeostasis by upregulating distal intestinal GLUT1 mediate decreasing TXNIP. a Body weight after metformin and STF-31 treatment. Mice did not exhibit any significant changes in body weight in response to metformin or co-treated metformin and STF-31 treatment. **b** Expression of various glucose transporter and gluconeogenesis enzyme in ileum and colon from vehicle and metformin treated mice. **c** Graph shows comparison of protein expression of Fig. 6f quantify with ImageJ. **d** Representative images of GLUT1 and TXNIP immunostaining of the ileum and colon from vehicle-treated, metformin-treated and cotreated with metformin and STF-31 C57BL/6 mice. scale bars indicate 100μm. All data are presented as the mean ± SEM. Data in b and c were analyzed using two-tailed Student's t-tests; *P < 0.05, **P < 0.01, ***P < 0.001.

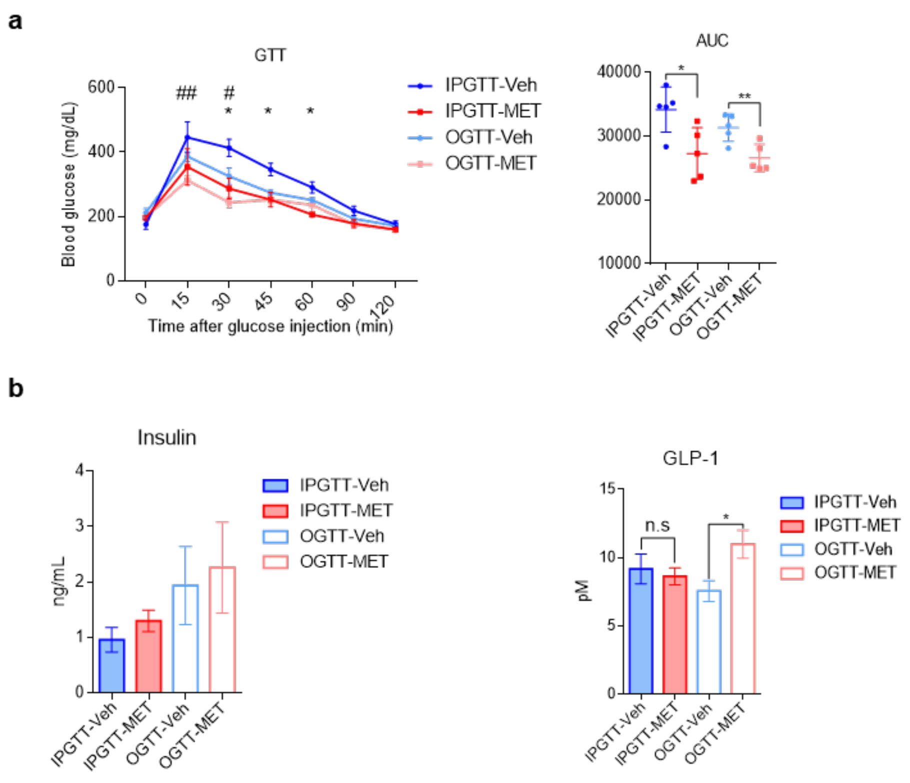

Supplementary Fig. 7. Evaluation of glucose and hormone responses in OGTT and IPGTT. **a** IPGTT and OGTT data for vehicle and metformin treated mice. Metformin-treated C57BL/6 mice (n = 5) exhibited improved glucose tolerance compared to vehicle-treated mice (n = 5) in both OGTT and IPGTT. No significant difference in total AUC was observed between OGTT and IPGTT. However, in OGTT, the glucose-lowering effect of metformin appeared earlier, with notable differences between groups observed at early time points (15–30 min). In contrast, IPGTT revealed group differences at later time points (30–60 min), indicating a delayed response consistent with reduced incretin involvement (analysis of variance multiple t-test for multiple comparison correction; *P < 0.05 use to compare IPGTT-Veh and IPGTT-MET, #P < 0.05, ##P < 0.01 use to compare OGTT-Veh and OGTT-MET). **b** Plasma insulin and GLP-1 levels following OGTT and IPGTT in vehicle- and metformin-treated mice. At 15 minutes post-glucose administration, plasma insulin levels tended to be higher in OGTT compared to IPGTT. Additionally, GLP-1 levels showed the highest tendency in the OGTT-metformin group, suggesting that the earlier glucose-lowering effect observed with OGTT may be associated with incretin hormone responses.

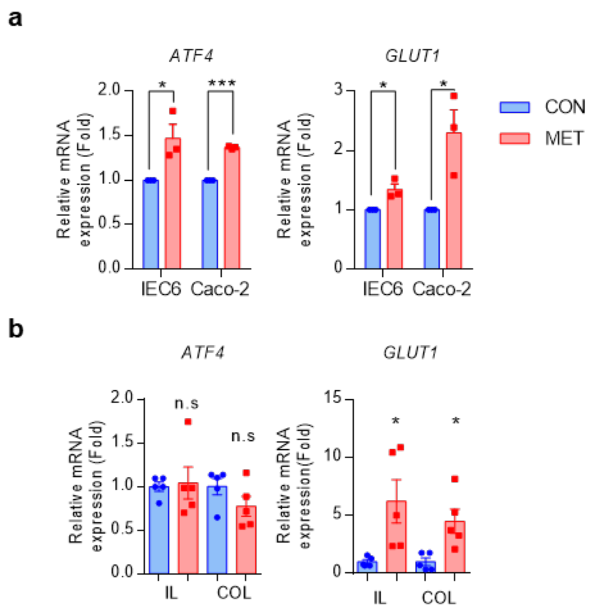

Supplementary Fig. 8. Metformin increased GLUT1 expression related to not ATF4.

a-b qRT-PCR result of ATF4 and GLUT1 **a** after metformin and phenformin treated in IEC6 and Caco-2 cell line. **b** Metformin(red) treated ileum and colon compare to Vehicle(blue). All data are presented as the mean ± SEM. Data in a and b were analyzed using two-tailed Student's t-tests; *P < 0.05, **P < 0.01, ***P < 0.001.

Supplementary Tables

Primer list table

Supplementary Table 1

No.	Oligo Name	5` - Oligo Seq - 3`
1	mGLUT1_F	CAGTTCGGCTATAACACTGGTG
2	mGLUT1_R	GCCCCCGACAGAGAAGATG
3	mGLUT2_F	ACTTGGAAGGATCAAAGCAATGT
4	mGLUT2_R	CAGTCCTGAAATTAGCCCACAA
5	mGLUT3_F	ATGGGGACAACGAAGGTGAC
6	mGLUT3_R	GTCTCAGGTGCATTGATGACTC
7	mGLUT4_F	GTGACTGGAACACTGGTCCTA
8	mGLUT4_R	CCAGCCACGTTGCATTGTAG
9	mGLUT5_F	CCAATATGGGTACAACGTAGCTG
10	mGLUT5_R	GCGTCAAGGTGAAGGACTCAATA
11	mSGLT1_F	ATGCGGCTGACATCTCAGTC
12	mSGLT1_R	ACCAAGGCGTTCCATTCAAAG
13	mHK2_F	TGATCGCCTGCTTATTCACGG
14	mHK2_R	AACCGCCTAGAAATCTCCAGA
15	mG6PC1_F	CGACTCGCTATCTCCAAGTGA
16	mG6PC1_R	GTTGAACCAGTCTCCGACCA
17	mPCK1_F	CTGCATAACGGTCTGGACTTC
18	mPCK1_R	CAGCAACTGCCCGTACTCC
19	mTXNIP_F	TCTTTTGAGGTGGTCTTCAACG

20	mTXNIP_R	GCTTTGACTCGGGTAACTTCACA
21	mATF4_F	CCTGAACAGCGAAGTGTTGG
22	mATF4_R	TGGAGAACCCATGAGGTTTCAA
23	mME1_F	AGAGCAGTGCTACAAGGTGACC
24	mME1_R	CCAAGAGCAACTCCAGGGAACA
25	mTXNRD1_F	GGGTCCTATGACTTCGACCTG
26	mTXNRD1_R	AGTCGGTGTGACAAAATCCAAG
27	mLDHA_F	ACGCAGACAAGGAGCAGTGGAA
28	mLDHA_R	ATGCTCTCAGCCAAGTCTGCCA
29	mBADH_F	GGCCGAGTGATTGCCACTT
30	mBADH_R	GGCCACTTTTCTTACTCCAGAG
31	mNNT_F	TCTCCACTCACCGCTGATGTCT
32	mNNT_R	AGCAGCAAGACTCTGAGAAGTTG
33	mNADK2_F	TGGCTGCGAGTAAAGTCCTG
34	mNADK2_R	CGAGAGAACCTCCGTAAGGC
35	mB2M_F	ACTGATACATACGCCTGCAGAGTT
36	mB2M_R	TCACATGTCTCGATCCCAGTAGA
37	rGLUT1_F	GTGCTCGGATCCCTGCAGTTCG
38	rGLUT1_R	GGGATGGACTCTCCATAGCGGTG
39	rGLUT2_F	TAGTCAGATTGCTGGCCTCAGCTT
40	rGLUT2_R	TTGCCCTGACTTCCTCTTCCAACT
41	rGLUT3_F	GCGCAGCCCTTCCGTTTTGC
42	rGLUT3_R	CGCTGGAGGATCTCCGTCGC

43	rGLUT4_F	GGTTTCACCTCCTGCTCTAA
44	rGLUT4_R	TGGCATGGGTTTCCAGTATG
45	rGLUT5_F	TCGCACTGGCACTGCAGAACA
46	rGLUT5_R	GCCCCACGGCGTGTCCTATG
47	rSGLT1_F	GACGGTGACGACGCTGATAG
48	rSGLT1_R	GCCTACGGAACTGGAAGCTG
49	rHK2_F	TGATCGCCTGCTTATTCACGG
50	rHK2_R	AACCGCCTAGAAATCTCCAGA
51	rG6PC1_F	AATGAACGTGCTCCACGACT
52	rG6PC1_R	CTGCCACCCAGAGGAGATTG
53	rPCK1_F	ATCCCCAAAAGTGGGCAGAG
54	rPCK1_R	TACATGGTGCGGCCTTTCAT
55	rTXNIP_F	CGAGTCAAAGCCGTCAGGAT
56	rTXNIP_R	TTCATAGCGCAAGTAGTCCAAGGT
57	rSNX27_F	GAGCAGGCGAGAAGGAATTG
58	rSNX27_R	GCTTAGAACACAGCTGCCTC
59	rTBC1D5_F	TTGAACAAAGGGCAAAGTCCG
60	rTBC1D5_R	GGCGATGTGGCTGAAAAAGG
61	rATF4_F	CTTCTCCAGGTGTTCCTCGT
62	rATF4_R	TGCTCAGCCCTCTTCTTCTG
63	rME1_F	TTGGGCGACCTTGGTTGTAA
64	rME1_R	CATTTTCTGTGCCCACGTCC
65	rTXNRD1_F	CCTCCTTGCTCATCCAAACAAAAA

66	rTXNRD1_R	AGTTGGTGTGACGAAGTCCA
67	rB2M_F	CCTCAACTGCTACGTGTCTCAG
68	rB2M_R	CCAGTCCTTGCTGAAGGACAG
69	hGLUT1_F	GGCCAAGAGTGTGCTAAAGAA
70	hGLUT1_R	ACAGCGTTGATGCCAGACAG
71	hGLUT2_F	GCCTGGTTCCTATGTATATCGGT
72	hGLUT2_R	GCCACAGATCATAATTGCCCAAG
73	hGLUT3_F	GCTGGGCATCGTTGTTGGA
74	hGLUT3_R	GCACTTTGTAGGATAGCAGGAAG
75	hGLUT4_F	ATCCTTGGACGATTCCTCATTGG
76	hGLUT4_R	CAGGTGAGTGGGAGCAATCT
77	hGLUT5_F	ACGTTGCTGTGGTCTGTAACC
78	hGLUT5_R	CATTAAGATCGCAGGCACGATA
79	hSGLT1_F	GCATCGCCTGGGTGCCCATT
80	hSGLT1_R	GCACCGTGCTGCTCTAGCCC
81	hHK2_F	GAGCCACCACTCACCCTACT
82	hHK2_R	CCAGGCATTCGGCAATGTG
83	hG6PC_F	ATTGCGGTTGCTGAGACTTT
84	hG6PC_R	ATCCAATGGCGAAACTGAAC
85	hPCK1_F	GAGAAAGCGTTCAATGCCAG
86	hPCK1_R	ATGCCGATCTTTGACAGAGG
87	hTXNIP_F	GGTCTTTAACGACCCTGAAAAGG
88	hTXNIP_R	ACACGAGTAACTTCACACACCT

89	hTBC1D5_F	CCTCAGCCTGGGTTTAGTAGA
90	hTBC1D5_R	CCGAGACAGGTCTGGTAGTTA
91	hSNX27_F	CAAGTCCGAGTCCGGCTAC
92	hSNX27_R	CCTGCTCGAATCAGGTCCA
93	hATF4_F	ATGACCGAAATGAGCTTCCTG
94	hATF4_R	GCTGGAGAACCCATGAGGT
95	hME1_F	GGGAGACCTTGGCTGTAATGG
96	hME1_R	TTCGGTTCCCACATCCAGAAT
97	hTXNRD1_F	GGTTCCACTGTCAAGTGTGACT
98	hTXNRD1_R	TCCCCAAACTTCTCCACAGC
99	hB2M_F	GAGTATGCCTGCCGTGTGAA
100	hB2M_R	TGCGGCATCTTCAAACCTCC

Supplementary Methods

Data processing and analysis

Paired-end sequencing reads were generated on the Illumina sequencing NovaSeq platform. Before starting the analysis, Trimmomatic v0.38 was used to remove adapter sequences and trim bases with poor base quality. The cleaned reads were aligned to the Mus musculus (mm10) using HISAT v2.1.0 (KIM et al, 2015), based on the HISAT and Bowtie2 implementations. The reference genome sequence and gene annotation data were downloaded from the NCBI Genome assembly and NCBI RefSeq database respectively. Aligned data (SAM file format) were sorted and indexed using SAMtools v 1.9. After alignment, the transcripts were assembled and quantified using StringTie v2.1.3b (Pertea, Mihaela, et al., 2015, 2016). Gene-level and Transcript-level quantification were calculated as raw read count, FPKM (Fragments Per Kilobase of transcript per Million mapped reads), and TPM (Transcripts Per Million).

Differential gene expression analysis

The relative abundances of gene were measured in FPKM (Fragments Per Kilobase of exon per Million fragments mapped) using StringTie [1-4]. We performed the statistical analysis to find differentially expressed genes using the estimates of abundances for each gene in samples. Genes with one more than zeroed FPKM values in the samples were excluded. To facilitate log2 transformation, 1 was added to each FPKM value of filtered genes. Filtered data were log2-transformed and subjected to quantile normalization. Statistical significance of the differential expression data was determined using an independent t-test and fold change in which the null hypothesis was that no difference exists among groups. False discovery rate (FDR) was controlled by adjusting p value using Benjamini-Hochberg algorithm. For the DEG set, hierarchical clustering analysis was performed using complete linkage and Euclidean distance as a measure of similarity. Gene-enrichment and functional annotation analysis and pathway analysis for significant gene lists were performed based on Gene Ontology (www.geneontology.org/). All data analysis and visualization of differentially expressed genes was conducted using R 3.6.1 (www.r-project.org). The data discussed in this publication have been deposited in NCBI's Gene Expression Omnibus (Edgar et al., 2002) and are accessible through GEO Series accession number GSE271623.

Supplementary References

1. Kim, D., Langmead, B. & Salzberg, S. L. HISAT: a fast spliced aligner with low memory requirements. *Nat Methods* **12**, 357-360 (2015).
2. Pertea, M., Pertea, G. M., Antonescu, C. M., Chang, T. C., Mendell, J. T. & Salzberg, S. L. StringTie enables improved reconstruction of a transcriptome from RNA-seq reads. *Nat Biotechnol* **33**, 290-295 (2015).
3. Pertea, M., Kim, D., Pertea, G. M., Leek, J. T. & Salzberg, S. L. Transcript-level expression analysis of RNA-seq experiments with HISAT, StringTie and Ballgown. *Nat Protoc* **11**, 1650-1667 (2016).
4. Raudvere, U. et al. g:Profiler: a web server for functional enrichment analysis and conversions of gene lists (2019 update). *Nucleic Acids Res* **47**, W191-w198 (2019).
